# Proteome profiling in the aorta and kidney of type 1 diabetic rats

**DOI:** 10.1371/journal.pone.0187752

**Published:** 2017-11-09

**Authors:** Moustafa Al Hariri, Mohamad Elmedawar, Rui Zhu, Miran A. Jaffa, Jingfu Zhao, Parvin Mirzaei, Adnan Ahmed, Firas Kobeissy, Fuad N. Ziyadeh, Yehia Mechref, Ayad A. Jaffa

**Affiliations:** 1 Department of Biochemistry and Molecular Genetics, Faculty of Medicine, American University of Beirut, Beirut, Lebanon; 2 Department of Chemistry and Biochemistry, Texas Tech University, Memorial Circle & Boston, Lubbock, Texas, United States of America; 3 Epidemiology and Population Health Department, Faculty of Health Sciences, American University of Beirut, Beirut, Lebanon; 4 Center for Biotechnology & Genomics, Texas Tech University, Canton & Main, Experimental Sciences building, Lubbock, Texas, United States of America; 5 Department of Internal Medicine, Faculty of Medicine, American University of Beirut, Beirut, Lebanon; Max Delbruck Centrum fur Molekulare Medizin Berlin Buch, GERMANY

## Abstract

Diabetes is associated with a number of metabolic and cardiovascular risk factors that contribute to a high rate of microvascular and macrovascular complications. The risk factors and mechanisms that contribute to the development of micro- and macrovascular disease in diabetes are not fully explained. In this study, we employed mass spectrometric analysis using tandem LC-MS/MS to generate a proteomic profile of protein abundance and post-translational modifications (PTM) in the aorta and kidney of diabetic rats. In addition, systems biology analyses were employed to identify key protein markers that can provide insights into molecular pathways and processes that are differentially regulated in the aorta and kidney of type 1 diabetic rats. Our results indicated that 188 (111 downregulated and 77 upregulated) proteins were significantly identified in the aorta of diabetic rats compared to normal controls. A total of 223 (109 downregulated and 114 upregulated) proteins were significantly identified in the kidney of diabetic rats compared to normal controls. When the protein profiles from the kidney and aorta of diabetic and control rats were analyzed by principal component analysis, a distinct separation of the groups was observed. In addition, diabetes resulted in a significant increase in PTM (oxidation, phosphorylation, and acetylation) of proteins in the kidney and aorta and this effect was partially reversed by insulin treatment. Ingenuity pathway analysis performed on the list of differentially expressed proteins depicted mitochondrial dysfunction, oxidative phosphorylation and acute phase response signaling to be among the altered canonical pathways by diabetes in both tissues. The findings of the present study provide a global proteomics view of markers that highlight the mechanisms and putative processes that modulate renal and vascular injury in diabetes.

## Introduction

Diabetes mellitus is a worldwide health burden manifested through hyperglycemia accompanied with insulin deficiency or resistance [1,2]. Hyperglycemia causes irreversible damage to blood vessels and highly vascularized organs at the microvascular and macrovascular levels, accounting for the highest mortality in diabetic patients, which render diabetes mellitus as an independent risk factor for Cardiovascular Diseases (CVD) and Chronic Kidney Disease (CKD) [3,4]. The diabetes-induced lesions at the microvascular level of the renal glomeruli result in diabetic nephropathy (DN), which constitutes the most recurrent and serious complication of diabetes mellitus [5]. On the other hand, the lesions at the macrovascular level lead to diabetes-induced atherosclerotic pathophysiology [6,7]. It has been shown that poor control of hyperglycemia at the early stages of diabetes would accelerate the incidence and progression of vascular and renal complications. Outcomes from The Diabetes Control and Complications Trial (DCCT) [8] and the Epidemiology of Diabetes Intervention and Complications (EDIC) [9] have proven that the primary modifiable risk factor for the long-term vascular and renal complications of T1DM is hyperglycemia [10].

Despite the focus on identifying the mediators of the disease progression in diabetes mellitus, the exact mechanisms for the cardiovascular and renal complications of T1DM are still unclear. Many studies have reported that endothelial dysfunction, oxidative stress, advanced glycation end products, a decrease in nitric oxide production and bioavailability, and deposition of fibrotic proteins are involved in the initiation or development of CVD and CKD [11–16]. In this study, we aimed at identifying the global protein changes in response to T1DM-induced hyperglycemia in the aorta and kidney, by employing Liquid chromatography-tandem mass spectrometry (LC-MS/MS) technique to comparatively quantitate the expression of different proteins among the different conditions, and to check the intensities of three different post-translational modifications, namely acetylation, phosphorylation and oxidation. In addition, systems biology analysis (Ingenuity Pathway Analysis, IPA) was used to model the effects of diabetes on different pathways in the two organs, to identify biological processes that are modified by the exposure conditions [17–19]. This approach allows the identification of possible novel biomarkers and development of new mechanisms aimed at defining the interplay of multiple biological pathways involved in the etiology of renal and vascular disease in diabetes.

## Methods

### Induction of diabetes

A total of 9 rats were used in the study divided into three groups with 3 rats in each group. Non-diabetic control n = 3, diabetic n = 3 and insulin treated diabetic, n = 3. The initial body weight of the rats used in the study were between 250–275g and were 8 weeks of age. Rats were housed two to three per cage in a light- and temperature-controlled room and had free access to food and water. Diabetes was induced by a single intravenous injection of streptozotocin (STZ), 65 mg/kg body weight through the tail vein. After 24 h, diabetes was confirmed in STZ-treated rats by tail vein plasma glucose levels. Glucose levels and body weights were measured at predetermined intervals to characterize the diabetic state. The insulin treated diabetic rats were treated twice daily with subcutaneous injections of insulin (3U, HUMULIN N) for 4 weeks, two weeks after induction of diabetes. At the end of the study period the rats were sacrificed by CO_2_ euthanasia followed by harvesting of tissues (kidneys and aorta). Both kidneys were excised from the rats and the cortices were dissected out and used in our study. The cortices from the left kidney were immediately frozen for proteomic analysis and the cortices from the right kidneys were embedded in paraffin for further analysis using immunohistochemistry. The aorta was removed, denuded and cut into two sections. One half was immediately frozen for proteomic analysis and the other half was embedded in paraffin for further analysis using immunohistochemistry. All animal experiments were approved by the Institutional Animal Care and Use Committee (IACUC) at the American University of Beirut and conducted in accordance with the guidelines of the Animal Care Facility and IACUC.

### Extraction and tryptic digestion of proteins

Denuded aorta and kidney cortices from control, diabetic and insulin-treated diabetic rats were homogenized using beads beater (Beadbug microtube homogenizer, Benchmark Scientific, Edison, NJ) followed by sonication on ice for 30 min and centrifugation at 14,800 rpm for 10 min. The resulting supernatant was diluted 10x with 50 mM ammonium bicarbonate (ABC) buffer, and the protein concentration was determined by BCA protein assay kit (Thermo Scientific/Pierce, Rockford, IL) according to the user’s manual.

Aliquots of 10μg extracted proteins from each sample were denatured at 80°C for 10 min followed by adding 200 mM dithiothreitol (DTT) and incubating at 60°C for 45 min. The resulting reduced proteins were then alkylated by adding 2–6μl Iodoacetamide (IAA, 200 mM solution) and incubating at 37°C in the dark for 45 min. Excessive IAA was quenched by additional DTT and incubating at 37°C for 30 min. Trypsin (Promega, Madison, WI) was added at a ratio of 1:25 (enzyme: proteins, w/w) into samples and incubated at 37°C for 18 hours followed by addition of formic acid at a final concentration of 0.5% to quench the enzymatic reaction. Samples were then centrifuged at 14,800 rpm for 10 min, and the resulting supernatant containing the digested peptides were dried and then resuspended in an aqueous solution containing 2% acetonitrile (ACN) and 0.1% formic acid (FA) for Liquid Chromatography-Mass Spectroscopy/Mass Spectroscopy (LC-MS/MS) analysis.

### Liquid Chromatography-Mass Spectroscopy/Mass Spectroscopy analysis

Aliquots (1μg) of tryptic digested samples were subjected to Liquid chromatography-electrospray ionization-tandem mass spectrometry (LC-ESI-MS/MS) analysis. The LC-MS/MS data was acquired using a Dionex Ultimate 3000 nano-LC system (Thermo Scientific, San Jose, CA) interfaced to an LTQ Orbitrap Velos mass spectrometer (Thermo Scientific, San Jose, CA) equipped with a nano-ESI source. Injected peptides were first purified on-line at a flow rate of 3 μl/min using a C18 Acclaim PepMap 100 trap column (75 μm I.D. x 2 cm, 3 μm particle sizes, 100 Å pore sizes, Thermo Scientific, San Jose, CA). Peptides were separated on a C18 Acclaim PepMap RSLC column (75 μm I.D. x 15 cm, 2 μm particle sizes, 100 Å pore sizes, Thermo Scientific, San Jose, CA). The column temperature was maintained at 29.5°C and the flow rate was 350 nl/min during the separation. The mobile phase consisted of solution A (97.9% water/2% ACN/0.1% FA) and solution B (99.9% ACN/0.1% FA). The separation of peptides was achieved by following gradient of solution B: 5% over 10 min, 5%-20% over 55 min, 20–30% over 25 min, 30–50% over 20 min, 50%-80% over 1 min, 80% over 4 min, 80%-5% over 1 min and 5% over 4 min. Data-dependent acquisition mode with two scan events was employed for MS/MS analysis. The first scan event was a full MS scan of 400–2000 *m/z* at a mass resolution of 15,000. In the second scan event, 10 most intense ions detected in the first scan event were selected with an isolation width of 3.0 m/z to perform CID MS/MS. The normalized collision energy (CE) was set to 35%, and an activation Q value was 0.250. The dynamic exclusion was set to have repeat count of 2, repeat duration of 30s, exclusion list size 200 and exclusion duration of 90s.

### Post-Translation modification analysis

The raw data obtained from LC-MS/MS analysis were processed with the MaxQuant software version 1.5.4.1. Database search was performed against UniProtKB/Swiss-Prot rat database (35842 entries). The search included cysteine carbamidomethylation as a fixed modification and multiple variable modifications, including methionine oxidation, phosphorylation of serine, threonine and tyrosine, in addition to acetylation of protein N-terminal, histidine and lysine. Corresponding with experimental procedures, trypsin was specified as the proteolysis enzyme and a maximum of 2 miscleavages were allowed. For identification, the peptide precursor mass tolerance was 20 pm in the first search and 4.5 pm in the main search. As for fragments matching, a deviation of 0.5 Da was allowed. Only peptides with a minimum length of 7 amino acids were considered for identification. The false discovery rate (FDR) was set to be 0.01 at both peptide and protein levels. The minimum ratio count was set as two to determine the intensities of proteins. Both unique and razor peptides were considered for quantification. Data generated by MaxQuant were further interpreted using Perseus. Filters were applied to eliminate reverse hits and common contaminants for peptide and protein identification, and an additional filter for protein was only identified by sites. Only proteins that were detected in at least 2 replicates of at least one sample group were reported.

### Systems biology assessment

IPA software (Ingenuity^®^ Systems, Version 33559992) was employed to examine functional correlations within the different treatment groups. IPA is a powerful tool that is widely used in the omics field to suggest/predict the effects of specific conditions or drugs on the biological outcomes [20–22]. Data sets containing protein identifiers (UniProt-KB) and corresponding expression values (Log2 [Fold change]) of different comparative groups were uploaded. The comparative groups were as follows: diabetic vs. control to analyze the effect of diabetes compared to time-matched control, insulin-treated diabetic vs. control to analyze the effect of insulin treatment compared to time-matched control, and insulin-treated diabetic vs. diabetic to analyze the effect of insulin treatment compared to the diabetic group. Each protein identifier was mapped to its corresponding protein object in the Ingenuity Pathways Knowledge Base. All mapped proteins were differentially expressed with p < 0.05 and overlaid onto global molecular networks developed from information contained in the knowledge base. Networks were then algorithmically generated based on their connectivity. Networks were “named” on the most prevalent functional group(s) present. Canonical pathways, Diseases and Bio Functions, and Ingenuity Tox List tools were overlaid on the networks.

### Statistical analysis and LC-MS/MS data analysis

Our analysis was initiated by conducting simple descriptive statistics on the overall mean for every group (control, diabetes, and diabetes with insulin) as well as at each time point. Normality of the data was assessed graphically using Q-Q plots that determines whether the data follows the normal distribution as well as numerically using Shapiro-Wilk test for normality. We conducted first our analysis at a cross sectional level using the nonparametric Kruskal-Wallis one way ANOVA to test for group effect and reported the P-value for Bonferroni correction for multiple tests between the groups to identify the groups that differed at each time point. Then we carried out longitudinal analysis that accounts for the correlation between the repeated measures on the same rat and adjust for time effects. In this regard we implemented an advanced approach of longitudinal analysis using generalized linear mixed models (GLMM). We accounted for the correlation in the repeated measures that pertain to the same rat using random intercepts for each rat specified in GLMM. Data are expressed as mean ± SE and significance was considered at p<0.05.

LC-MS/MS data were used to generate mascot generic format file (*.mgf) by Proteome Discover version 1.2 software (Thermo Scientific, San Jose, CA) then searched using SwissProt database (Rattus) in MaxQuant version 2.4 (Matrix Science Inc., Boston, MA). Iodoacetamide modification of cysteine was set as a fixed modification, while oxidation of methionine was set as a variable modification. An m/z tolerance of 5 ppm was set for the identification of peptides with maximum 2 missed cleavages. Also, tandem MS ion tolerance was set within 0.8 Da with label-free quantification. Scaffold Q+ (Proteome Software, Portland, OR) was employed for spectral counts quantitation. The normalized intensity of target peptides corresponding to each candidate protein was summed up to represent the abundance of the certain protein. Student’s t-tests were performed to determine the significant proteins in the comparison between every two sample groups with a criteria of p-value < 0.05.

## Results

### Characteristics of the diabetic state

**Fig 1:**
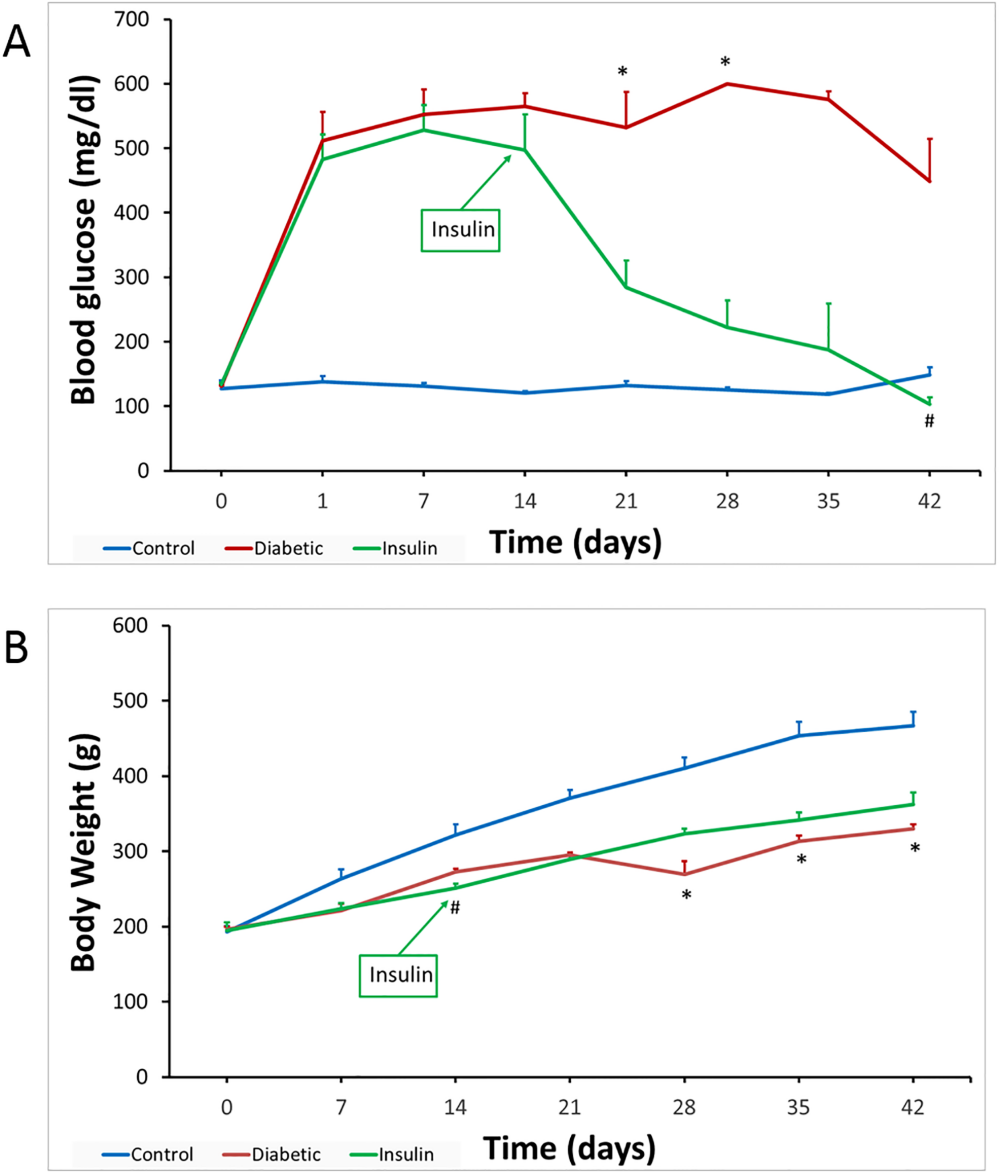
Plasma glucose levels (A) and body weights (B) in control, diabetic and insulin-treated diabetic rats. **(A)** Plasma glucose levels were significantly increased one day after STZ injection in both diabetic groups compared to controls. **(B)** Initial body weights were not significantly different between diabetic and control rats. The blue line is for Control rats, the red line for Diabetic rats, and the green line is for Insulin-treated diabetic rats. Cross-sectional analysis using non-parametric Kruskal-Wallis followed by Bonferroni correction was employed to assess the difference between the groups at each time point. * represents a significant difference between diabetes and control groups with p<0.03, and # represents a significant difference between insulin-treated diabetes and diabetes groups with p = 0.02.

Plasma glucose levels and body weights of all three groups of rats over the duration of the study period are shown in Fig 1A and 1B, respectively. Our analysis indicated that both glucose and weight were not normally distributed with respective Shapiro-Wilk test P-values of <0.0001 and 0.04. The summary statistics conducted at a group level (control, diabetic, and insulin-treated diabetic rats) revealed that the group of diabetic rats had the highest glucose levels 489±32 mg/dl. The second highest group in plasma glucose was that of insulin-treated diabetic rats with average plasma glucose levels of 305±36 mg/dl. Controls had the lowest plasma glucose levels with 130±3 mg/dl. Our nonparametric data analysis conducted cross sectional at each time point indicated that there was a significant difference in plasma glucose levels between control and diabetic rats at weeks 3 and 4 with a Bonferroni adjusted p = 0.02 and 0.019, respectively, and between diabetic and insulin-treated diabetic rats at week 6 with a Bonferroni adjusted p = 0.02. To determine if the observed difference in the plasma glucose levels between the groups is of statistical significance and to account for the longitudinal nature of the data we implemented the repeated measure generalized linear mixed models with an intercept as the random effect. This random effect will account for the correlation between the glucose measures repeated for the same rat, referred to as within subject correlation. The outcome glucose was modeled as a function of group and week. In addition, to account for any potential interaction effect, we included in this model an interaction term between week and group. Our longitudinal analysis indicated that group had a significant effect on plasma glucose levels with overall p< 0.0001. In addition, a significant interaction between week and group was detected (p = 0.0002), hence this interaction term was left in the model to account for its effect modification on the relationship between the group and plasma glucose. Time had a non-significant effect on plasma glucose, indicating that time is not significantly affecting the levels of glucose. Accordingly, group is the main factor that is associating with plasma glucose. In specific, the diabetes group had a significantly different (higher) glucose levels compared to controls (p< 0.0001), and diabetes with insulin had also significant difference (higher) levels of glucose compared to controls (p = 0.0003). Diabetes and diabetes with insulin did not have a significant difference in glucose (p-value = 0.2689).

The body weight for the controls was 354 ±2 g and that for diabetes were 271±10 g and that for diabetes and insulin were 284±13g. Our nonparametric data analysis conducted at a cross sectional level on every week indicated that there was a significant difference in body weight between control and insulin-treated diabetic rats at weeks 2, with an adjusted Bonferroni p = 0.02 and between control and diabetic rats at weeks 4, 5, and 6 with an adjusted Bonferroni p = 0.02, 0.03 and 0.03 respectively. Our longitudinal analysis using the generalized linear mixed models with random effect on the intercept with lognormal distribution and unstructured variance-covariance matrix revealed that there is a significant incremental effect of time (weeks) on weight (p< 0.0001), and a significant interaction between time and group (p < 0.0001). However, body weight did not significantly differ between the 3 groups with overall P-value of 0.2507, Fig 1B.

### Aorta and kidney proteomic analysis

Comparative proteomic profiling of kidney and aorta protein abundance was done using LC-ESI-MS/MS followed by MaxQuant analysis of the generated protein spectra. Adopting this approach, 1128 distinct proteins were identified in the aorta of control and diabetic rats and 1290 distinct proteins were identified in the kidney of control and diabetic rats. The frequencies of log (base 2) -transformed intensities of proteins in each sample were plotted (Figure A in S1 File), showing that the intensity frequency follow normal distribution. The list of identified proteins generated by LC-MS/MS in the different groups are shown in the protein group data (S2 File).

### Effect of diabetes on proteome profile in the aorta and kidney

**Fig 2:**
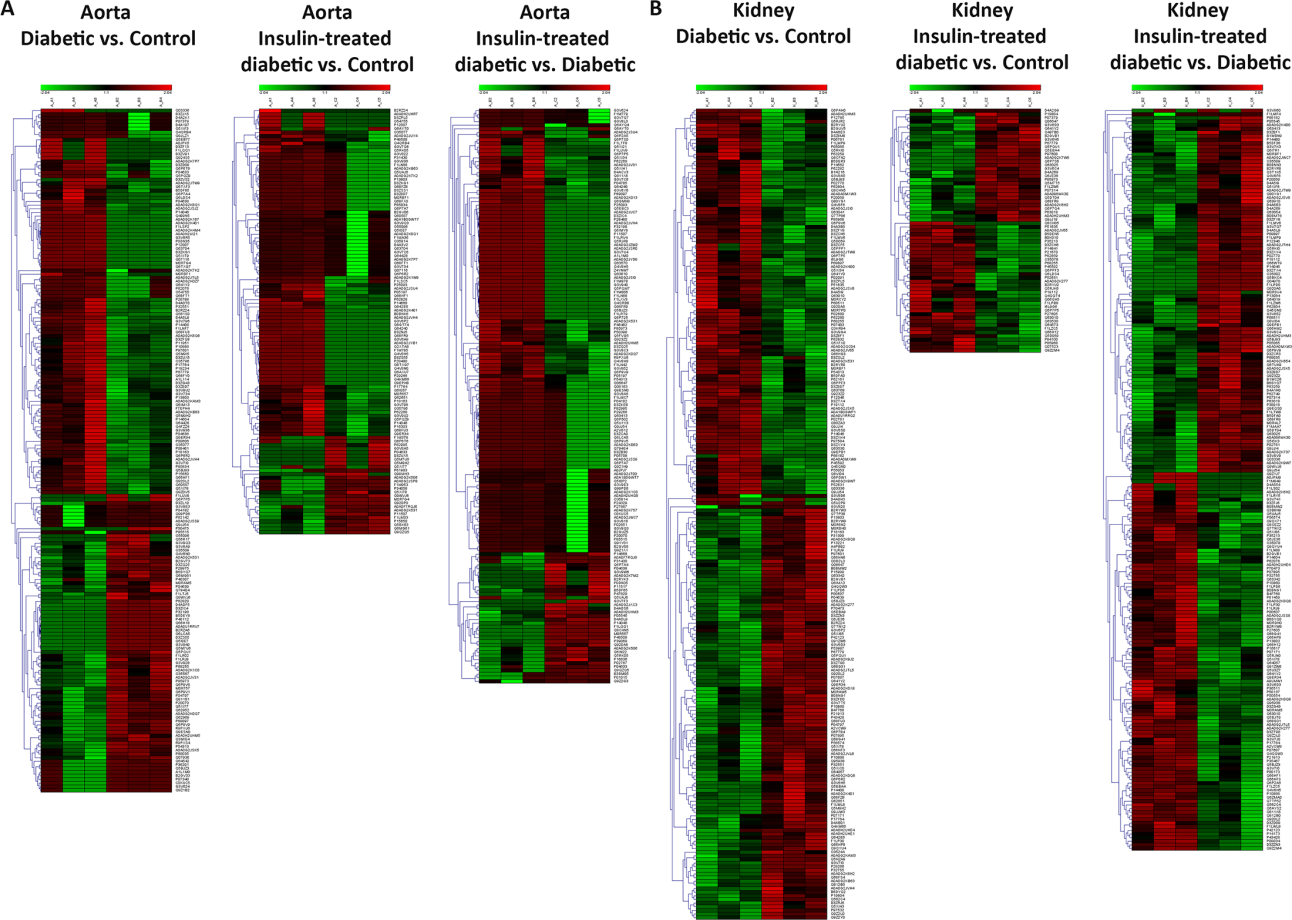
Hierarchical clustering (heat maps) of protein expression profiles in the aorta (A) and kidney (B) among the three groups of rats. The compared groups are Diabetic vs. Control samples, Insulin-treated diabetic vs. Control samples, and Insulin-treated diabetic vs. Diabetic samples. Green color represents downregulation of protein expression, whereas the red color represents upregulation of protein expression. Color intensity reflects the expression level of the proteins. The label on the right-hand side of the heat maps represents the accession number of the proteins.

The aorta and kidney responded to the diabetic state differently as evidenced by the proteomic profile in these tissues and as illustrated in the heat maps (Fig 2). Amid the 1128 identified proteins in the aorta, 188 (16.6%) were significantly modified by the diabetic state with p< 0.05. Of these, 111 (59%) proteins were significantly downregulated and 77 (41%) proteins were significantly upregulated (Table A in S1 File). Among the 1290 proteins identified in the kidney, 223 (17.3%) were significantly modified by the diabetic state with p< 0.05. Out of these, 109 (48.8%) proteins were significantly downregulated and 114 (51.1%) proteins were significantly upregulated (Table B in S1 File). A Venn diagram was generated to determine the common proteins modified by diabetes in the aorta and kidney. Only 12% of proteins that their expression was significantly modified by the diabetic state were common between the aorta and kidney. The list of common proteins is shown in Table C in S1 File, and included proteins related to carbon metabolism, citrate cycle (TCA cycle), metabolic pathways, microbial metabolism in diverse environments, fatty acid degradation, fatty acid metabolism and other pathways.

### Effect of diabetes on the expression levels of distinct oxidative stress enzymes

Diabetes resulted in the differential expression of a cluster of oxidative stress-related enzymes involved in hydrogen peroxide, electrophilic and superoxide detoxification in the aorta and kidney. In the aorta, diabetes-induced the protein expression levels of glutathione peroxidase 1 (3.22 fold, p = 0.041) and 3 (2.12 fold, p = 0.004), glutathione S-transferase Pi 1 (2.04 fold, p = 0.021) and Mu 3 (16.78 fold, p = 0.039), monoamine oxidase A (2.67 fold, p = 0.049) and reduced the expression of superoxide dismutase 1 (0.7 fold, p = 0.044) compared to levels in aorta of control normal rats (Table A in S1 File). Whereas in the kidney, diabetes-induced the protein expression levels of glutathione S-transferase Mu 1 (1.55 fold, p = 0.039) and Zeta 1 (1.86 fold, p = 0.005), peroxiredoxin 3 (1.36 fold, p = 0.001) and 6 (1.67 fold, p = 0.047) and superoxide dismutase 2 (1.24 fold, p = 0.018) compared to levels in kidney of control normal rats (Table B in S1 File).

### Effect of diabetes on matrix proteins in the aorta

Analysis of the proteomic abundance of proteins indicated that diabetes induced the expression levels of collagen type VI α6 (4.32 fold, p = 0.03), collagen type XVIII α1 (2.75 fold, p = 0.035), and fibulin 1 (4.12 fold, p = 0.013) compared to control tissues. In addition, diabetes induced the protein expression of integrin beta 1 (2.77 fold, p = 0.044), a focal adhesion protein that links the actin cytoskeleton to the extracellular matrix (Table A in S1 File).

### Effect of insulin treatment on proteome profile in the aorta and kidney

Treatment of diabetic rats with insulin for four weeks resulted in partial reversal of the expression profile of the proteins in aorta and kidney that were significantly modified by the diabetic state as shown in the heat maps Fig 2. Of the 188 proteins significantly modified by diabetes in the aorta, only 42 (22%) proteins responded to insulin treatment by normalizing the expression levels of these proteins (Table D in S1 File). Similarly, of the 223 proteins significantly modified by the diabetic state in the kidney, only 101 (45%) responded to insulin treatment by normalizing the expression levels of these proteins (Table E in S1 File).

### Effect of diabetes and insulin on kininogen and angiotensin-converting enzyme

Comparative analysis of the proteomic profile indicated that diabetes induced the protein expression of high molecular weight kininogen in the aorta (2.91 fold, p = 0.004) as well as in the kidney (4.61 fold, p = 0.012) compared to controls. It is of interest also to point here that insulin treatment resulted in increased expression of angiotensinogen levels by 5.89 fold (p = 0.025) and angiotensin-converting enzyme by 2.03 fold (p = 0.007) in the aorta.

### Principal component analysis (PCA)

**Fig 3:**
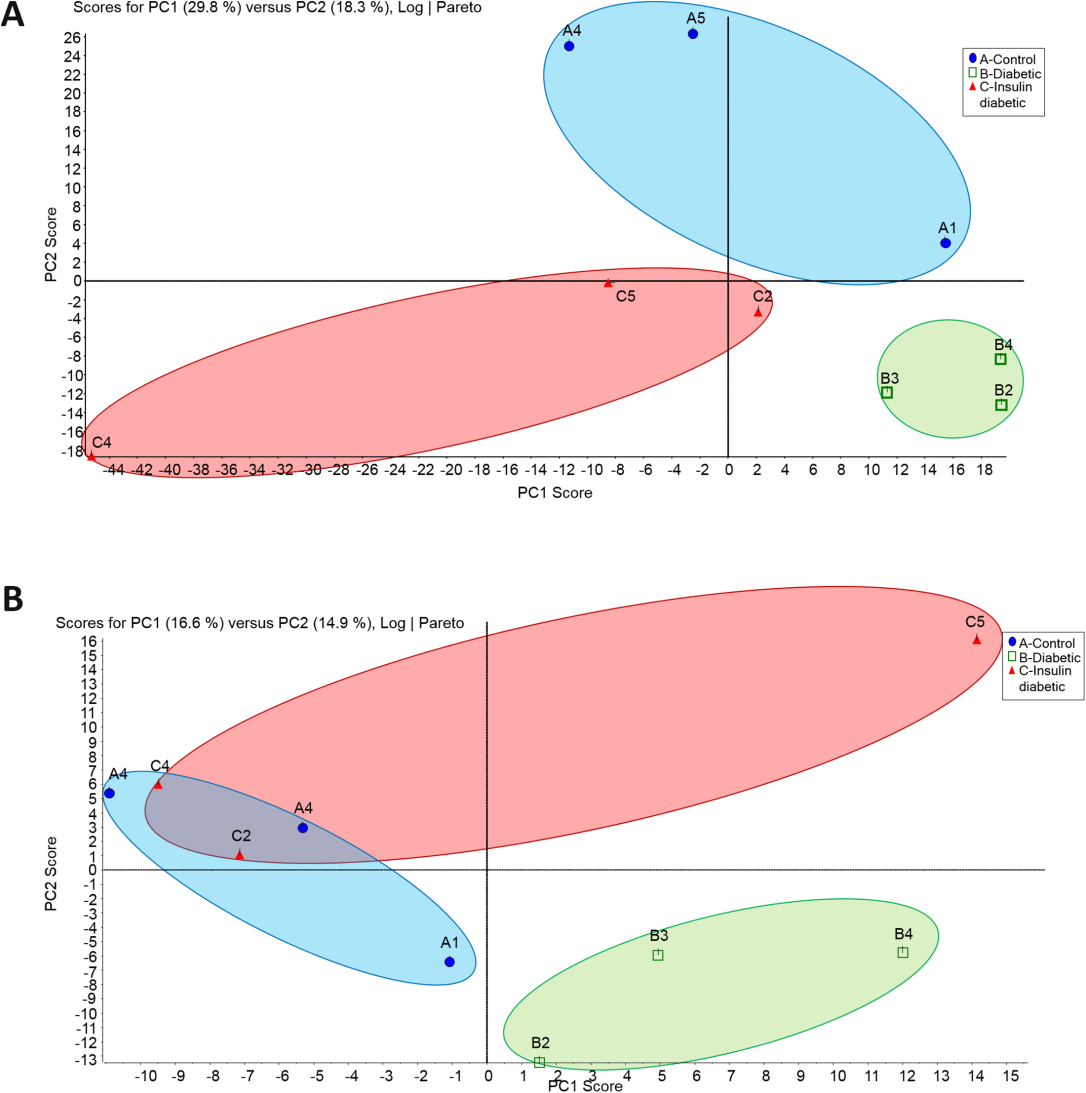
Principal component analysis (PCA) of the aorta (A) and the kidney (B) samples. The total normalized expression data of the proteins was used to depict the scatter plots of the first (X) and the second (Y) principal components. The numbering of the nodes is an identification of the samples. Abbreviation A (Control), B (Diabetic), and C (Insulin-treated Diabetic). Blue oval encircles the control samples, green oval encircles the diabetic samples, and red oval encircles the Insulin-treated diabetic samples.

PCA a derivative of multivariate data analysis, commonly used to reduce multidimensionality of large datasets and discern features that distinguish the different groups was applied to the proteomic profiles of the aorta and kidney. As shown in Fig 3A and 3B, PCA efficiently separated the groups, indicating that the proteomic profiles contain structure that is discernable even without considering the identity of individual factors. Importantly, the discriminatory power of the analysis held when considering the aorta and kidney proteome between the three different groups.

### Effect of diabetes on PTMs

**Fig 4:**
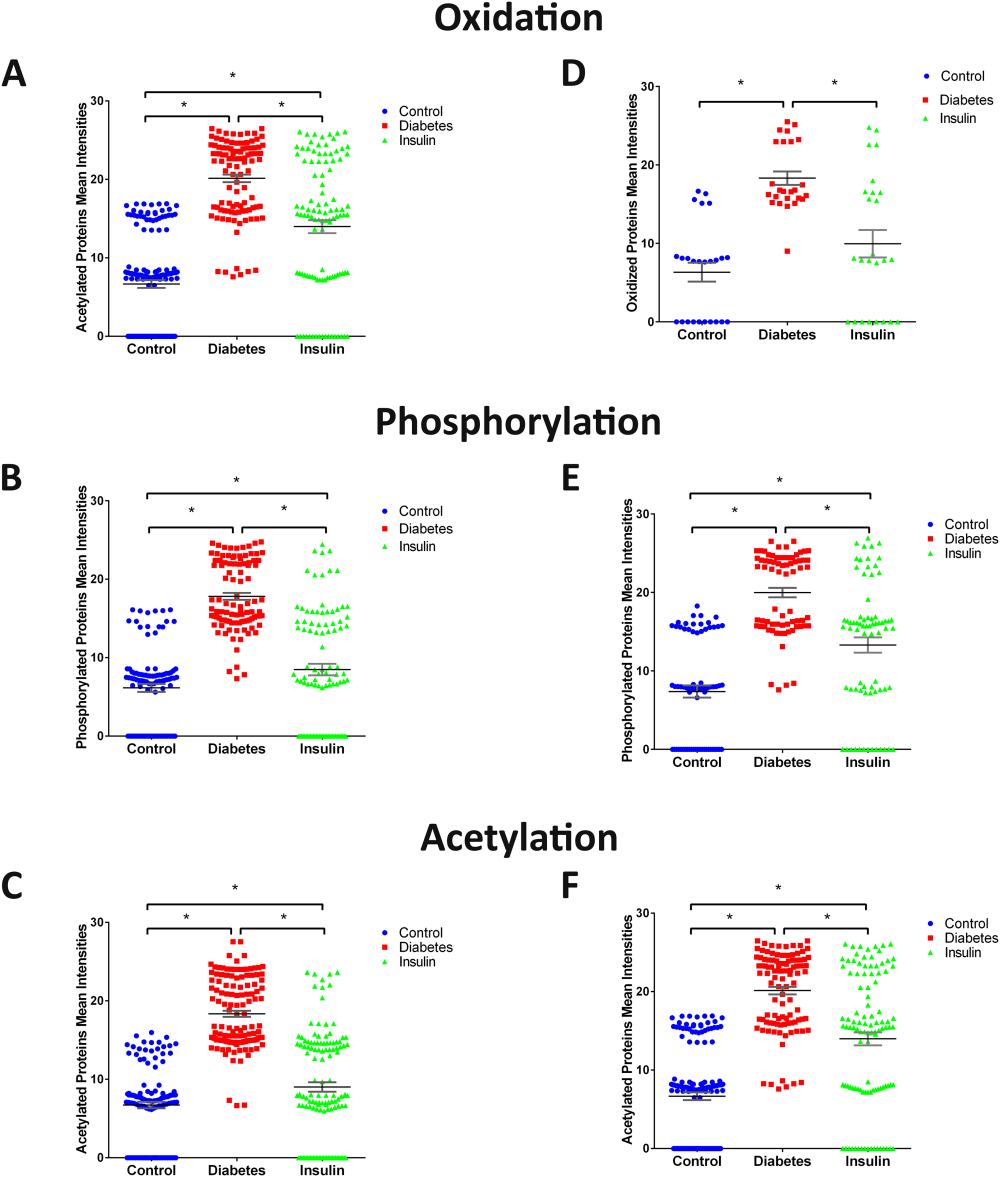
Scatter plot of the mean intensities of the three PTMs in the aorta (A, B, and C) and the kidney (D, E, and F) samples. A and C show the scatter of the oxidized proteins among the 3 different groups. B and E show the scatter of the phosphorylated proteins among the 3 different groups. C and F show the scatter of the acetylated proteins among the 3 different groups (* p<0.05).

Beyond protein abundance, LC-MS/MS also determined the intensity of proteins modified by oxidation, phosphorylation, and acetylation in the aorta and kidney of control, diabetic and insulin-treated diabetic rats. The increased intensity of the PTM proteins we observed was not due to the abundance of the specific proteins but rather to an effect of the diabetic state on the intensity of the modified PTM. The mean intensities of oxidized proteins in control aorta is 7.2 ± 0.93 compared to 18.1±0.71 in diabetic aorta (p< 0.001, n = 34) and 6.3±1.2 in control kidney compared to 18.3±0.85 in diabetic kidney (p< 0.001, n = 25). Treatment of diabetic rats with insulin significantly reduced the increased intensities of oxidized proteins in the aorta to 10.03±1.3 and kidney to 9.96±1.75 of diabetic rats to levels not significantly different from controls (Fig 4). The mean intensities of phosphorylated proteins in control aorta is 6.2±0.5 compared to 17.8±0.44 in the diabetic aorta (p<0.001, n = 100) and 7.4±0.77 in control kidney compared to 19.9±0.6 in diabetic kidney (p< 0.001, n = 72). Treatment of diabetic rats with insulin significantly reduced the increased intensities of phosphorylated proteins in the aorta to 8.49±0.73 and in the kidney to 13.3±0.97 compared to levels in diabetic rats (Fig 4). The mean intensities of acetylated proteins in control aorta is 6.7±0.42 compared to 18.4±0.39 in the diabetic aorta (p<0.001, n = 128) and 6.6±0.49 in control kidney compared to 20.1±0.48 in diabetic kidney (p<0.001, n = 105). Treatment of diabetic rats with insulin significantly reduced the increased intensities of acetylated proteins in the aorta to 9.02±0.6 and in the kidney to 13.99±0.82 compared to levels in diabetic rats (Fig 4).

### Pathway and network analysis of proteomic profiles

**Fig 5:**
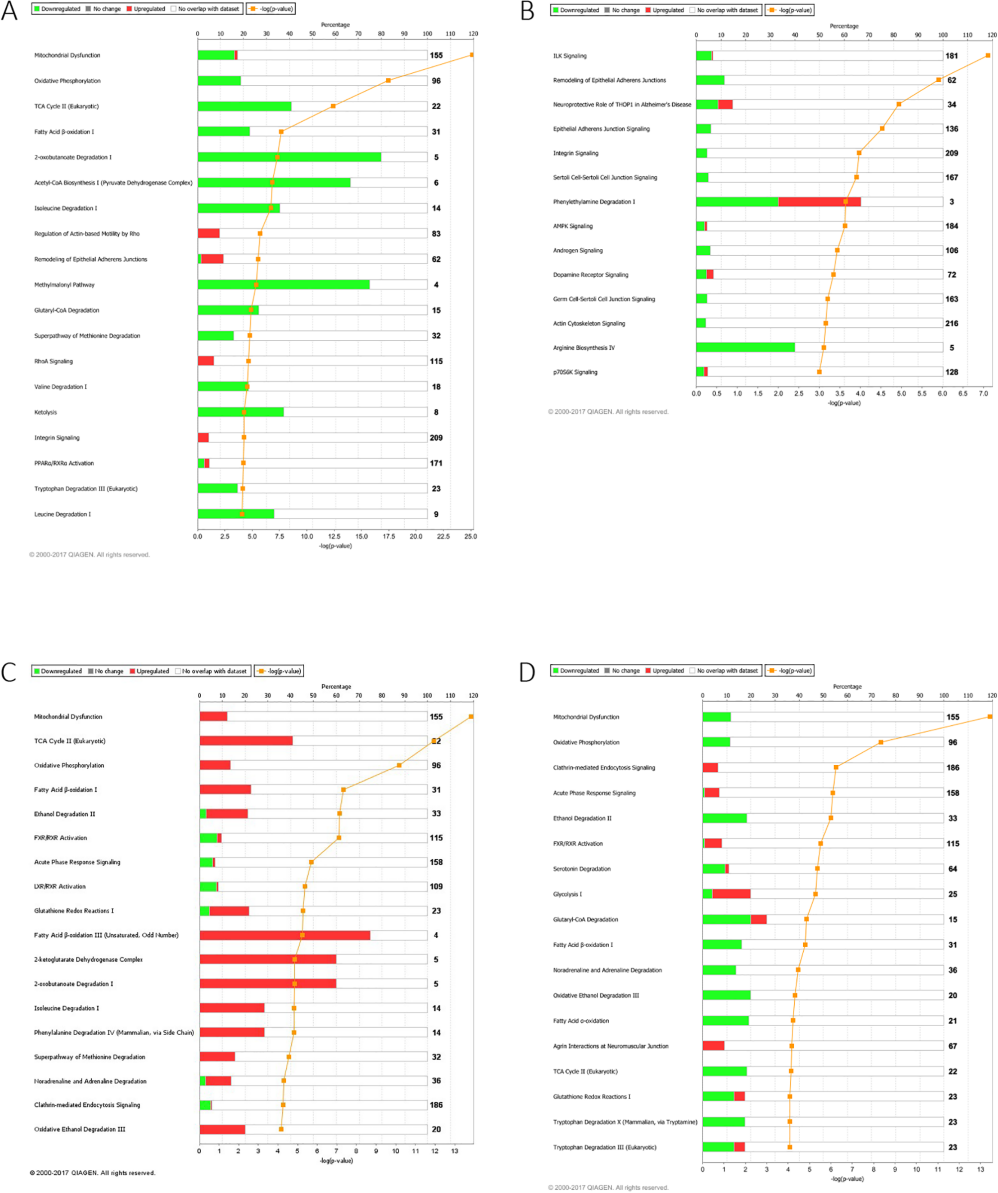
Canonical pathways of the comparative groups in the aorta and the kidney samples. **A:** top Canonical pathways related to proteins altered in the aorta of Diabetes vs. control samples. **B:** Top Canonical Pathways related to proteins altered in the aorta of Insulin-treated Diabetic vs Diabetic samples **C:** Top Canonical Pathways related to proteins altered in the kidney of Diabetic vs. Control samples. **D:** Top Canonical Pathways related to proteins altered in the kidney of Insulin-treated Diabetic vs. Diabetic samples. Bars show the total number of proteins identified in each pathway. The green color represents the downregulated proteins, and the red color represents the upregulated proteins.

The proteomic profiles of significantly modified proteins in the aorta and kidney were subjected to IPA analysis. The results shown in Fig 5A–5D offer a graphical representation of the altered canonical pathways of significantly upregulated and downregulated proteins within each tissue and between each group.

**Fig 6:**
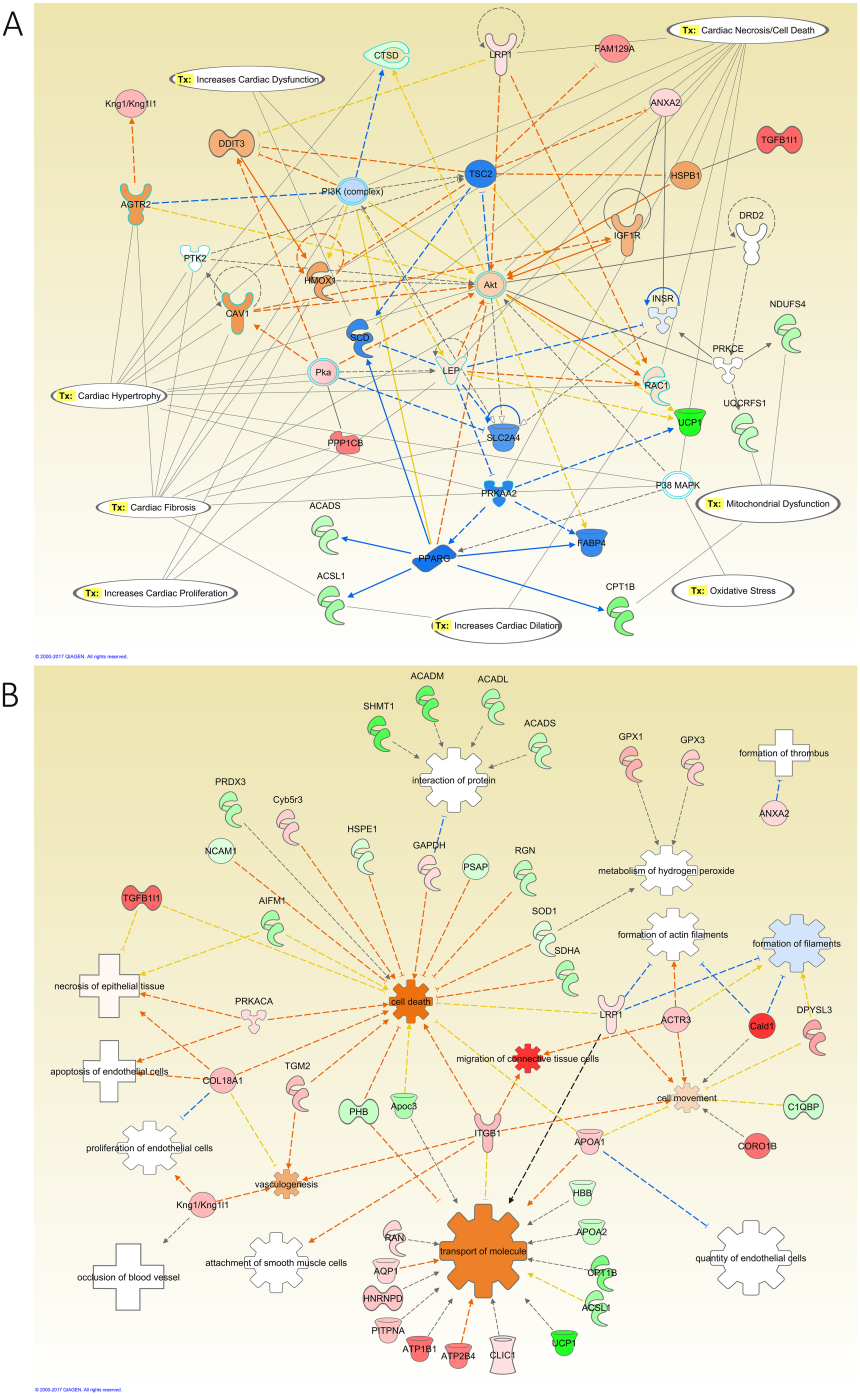
IPA network analysis of the modified proteins in the aorta of diabetic relative to control rats. **A:** The top diseases and functions related to the modified proteins are Lipid Metabolism, Molecular Transport, and Small Molecule Biochemistry. The top Toxicity and functions predicted by IPA related to the modified proteins are Cardiac Fibrosis, Cardiac Hypertrophy, Cardiac Necrosis/Cell Death, Increased Cardiac Dysfunction, Increased Cardiac Proliferation, Increased Cardiac Dilatation, Mitochondrial Dysfunction, and Oxidative Stress. The main regulated proteins connected in this network were Kng1, AKT, and Leptin. **B:** Diseases and Biological Functions predicted by IPA related to the differentially expressed proteins in this comparative group. The diseases related to these proteins are apoptosis of endothelial cells, formation of thrombus, necrosis of epithelial tissue, and occlusion of blood vessel. The color intensity of the nodes reflects the expression of the proteins. Green nodes are downregulated proteins, red nodes are upregulated proteins, white nodes are IPA predicted proteins with non-consistent activation pattern, blue nodes are IPA predicted proteins to be inhibited, and orange nodes are IPA predicted proteins to be activated.

Network analysis of aorta of diabetic rats compared to control (Fig 6A) depicted that kininogen (KNG1), transforming growth factor beta (TGFβ), and protein kinase A (PKA) were upregulated by the diabetic state and they were connected to the activation of angiotensin II receptor type 2 (AGTR2), Leptin (LEP), and heme oxygenase 1 (HOX1). In addition, network analysis showed that the modified proteins possessed many aorta toxicity related functions such as cardiac dysfunction, necrosis, fibrosis, hypertrophy, dilatation, proliferation, cell death, movement and migration of connective tissue (Fig 6A and 6B). To validate the proteomic analysis findings from the LC-MS/MS of the effects of diabetes on the expression of proteins in the aorta samples, we performed immunohistochemistry (IHC) staining for TGFβ. The results shown in Figure B in S1 File indicated an increase in the TGFβ (2.5±0.39-fold) staining in the aorta sections of the diabetic rats compared to the control. In addition, insulin supplementation showed a significant decrease in the staining of TGFβ (0.9±0.05-fold) compared to the diabetes group.

**Fig 7:**
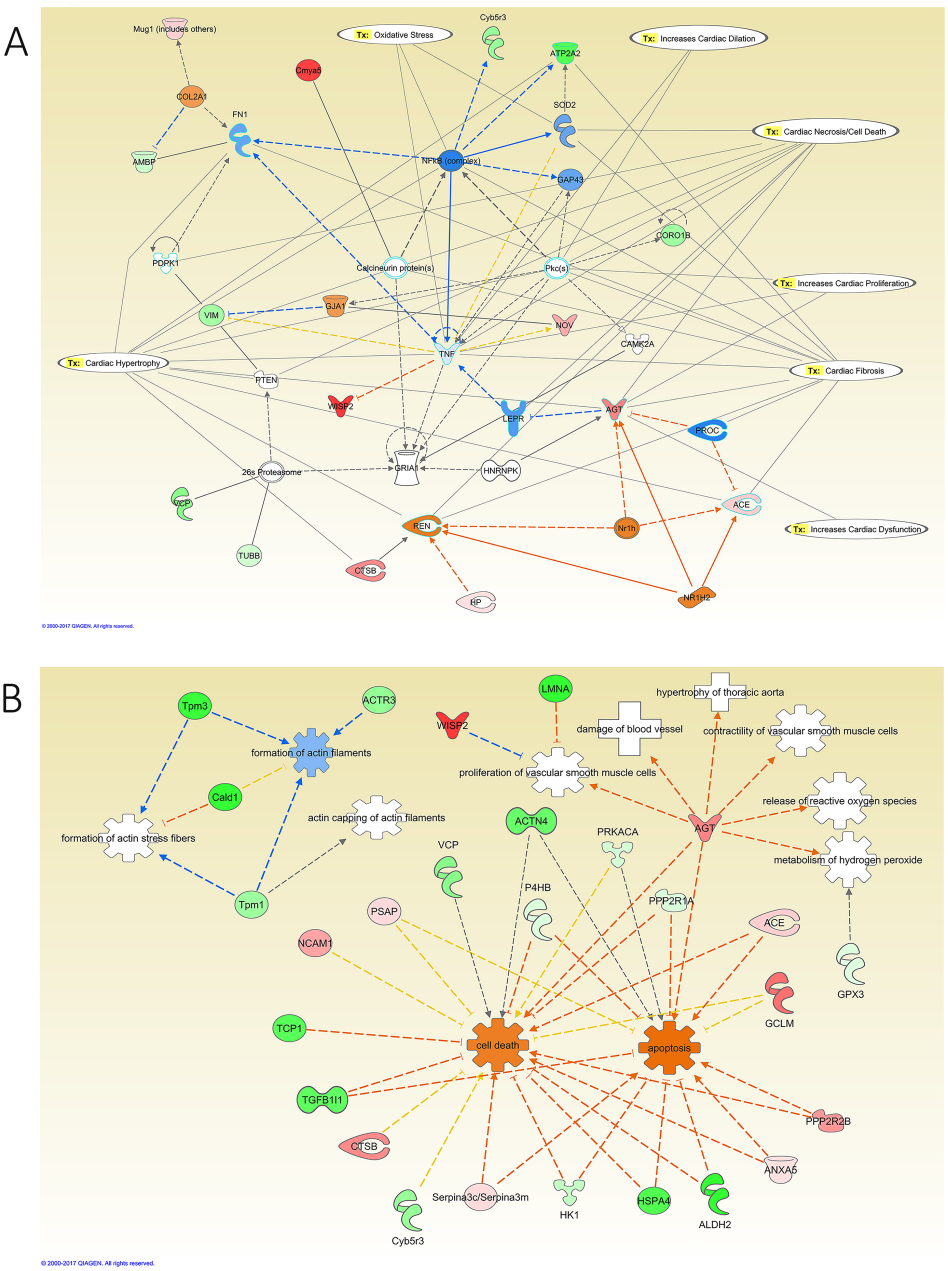
IPA network analysis of the modified proteins in the aorta of insulin-treated diabetic relative to diabetes rats. The top Toxicity and functions predicted by IPA related to the modified proteins are Cardiac Fibrosis, Hypertrophy, Necrosis/Cell Death, Oxidative Stress, Increased Cardiac Proliferation, Dilation, and Dysfunction. The main regulated proteins connected in this network are ACE, AGT, and TNF. **B:** Diseases and Biological Functions predicted by IPA related to the differentially expressed proteins in this comparative group. The main biological functions altered in this network are apoptosis, metabolism of hydrogen peroxide, and release of reactive oxygen species. The diseases related to these proteins are damage of blood vessel, and hypertrophy of thoracic aorta. The color intensity of the nodes reflects the expression of the proteins. Green nodes are downregulated proteins, red nodes are upregulated proteins, white nodes are IPA predicted proteins with non-consistent activation pattern, blue nodes are IPA predicted proteins to be inhibited, and orange nodes are IPA predicted proteins to be activated.

Network analysis of the effect of insulin treatment on the aorta of diabetic rats, showed that angiotensinogen (AGT), and angiotensin converting enzyme (ACE) were upregulated and connected to the inhibition of leptin receptor (LEPR), while alpha-1-microglobulin/bikunin precursor (AMBP) was downregulated and connected to the inhibition of fibronectin 1 (FN1). In addition, insulin altered oxidative stress through the upregulation of glutamate-cysteine ligase modified (GCLM), inhibition of tumor necrosis factor (TNF), nuclear factor kappa-light-chain-enhancer of activated B cells (NFκB), and superoxide dismutase 2 (SOD2), and activation of CYP (Fig 7A). Toxicity functions involved included cardiac dysfunction, necrosis, fibrosis, hypertrophy, dilatation, and proliferation. Additionally, the list of modified proteins showed activation of cell death, and apoptosis and inhibited the formation of actin filaments as biological functions (Fig 7B).

**Fig 8:**
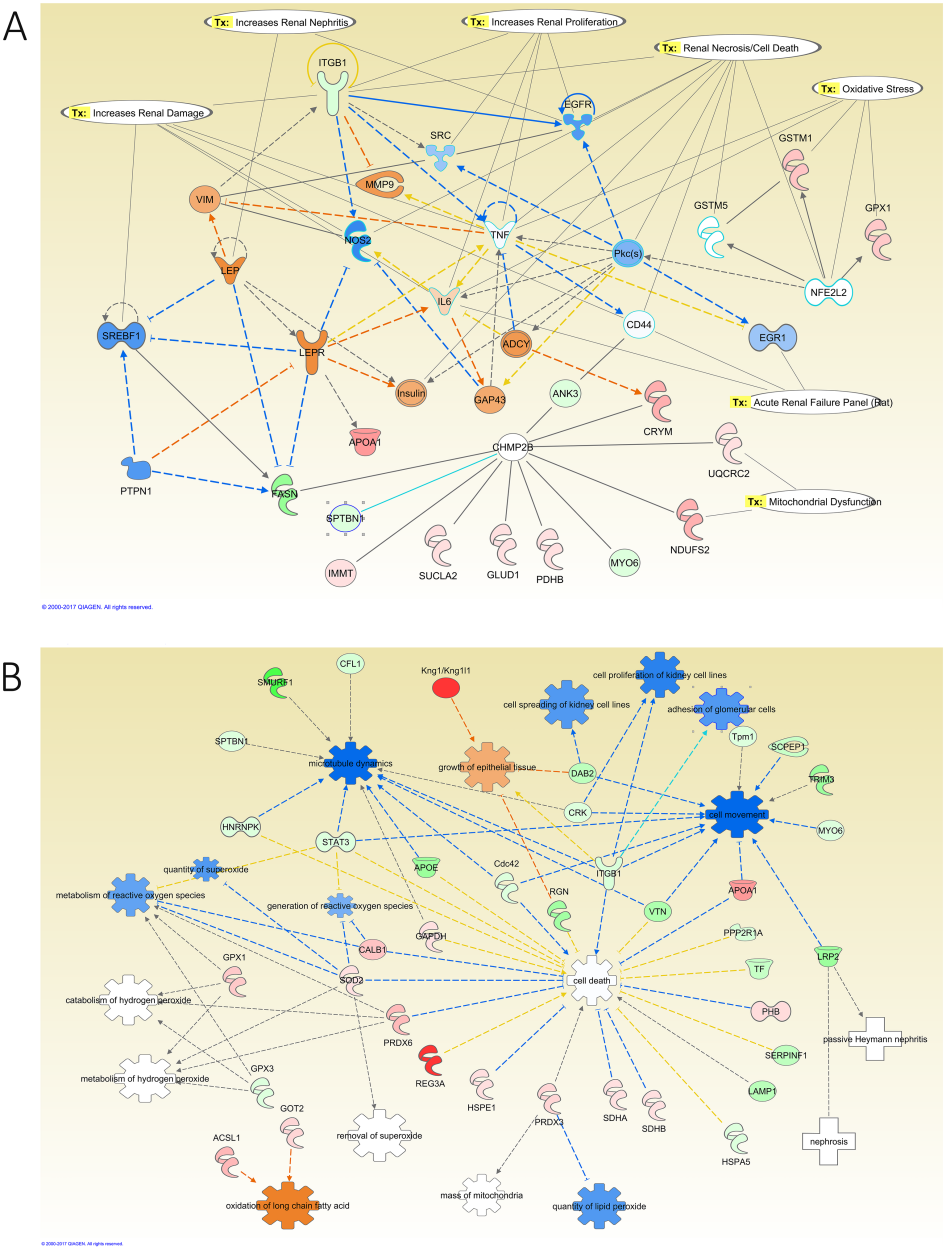
IPA network analysis of the modified proteins in the kidney of diabetic relative to control rats. **A:** The top Toxicity and functions predicted by IPA related to the modified proteins are Increase Renal Damage, Nephritis, Proliferation, Necrosis/Cell Death, Oxidative Stress, Acute Renal Failure Panel (Rat), and Mitochondrial Dysfunction. The main regulated proteins in this network are EGFR, LEP, LEPR, and TNF. **B:** Diseases and Biological Functions predicted by IPA related to the differentially expressed proteins in this comparative group. The main biological functions altered in this network are adhesion of glomerular cells, generation of reactive oxygen species, the growth of epithelial tissue, and metabolism of hydrogen peroxide and reactive oxygen species. The diseases related to these proteins are nephrosis, and passive Heymann nephritis. The color intensity of the nodes reflects the expression of the proteins. Green nodes are downregulated proteins, red nodes are upregulated proteins, white nodes are IPA predicted proteins with non-consistent activation pattern, blue nodes are IPA predicted proteins to be inhibited, and orange nodes are IPA predicted proteins to be activated.

Network analysis of the modified proteins in the kidney of diabetic rats revealed that the downregulation of integrin subunit beta 1 (ITGB1), Cofilin1 (CFL1), and fatty acid synthase (FASN) to be connected with the activation of leptin (LEP), LEPR, matrix metallopeptidase 9 (MMP9), and interleukin 6 (IL6). Diabetes promoted oxidative stress in the kidney through the downregulation of signal transducer and activator of transcription 3 (STAT3), the upregulation of SOD2, glutathione peroxidase 1 (GPX1), and glutathione S-transferase mu 1 (GSTM1), in addition to the activation of IL6 and the inhibition of NFκB activity (Fig 8A). IPA also identified toxicity functions of the detected proteins from the kidneys. These included renal damage, nephritis, necrosis, proliferation, and renal failure. Furthermore, the modified proteins in the kidney of the diabetic relative to control group showed activation of biological functions such as the activation of growth of epithelial tissue and oxidation of long chain fatty acid, and the inhibition of the adhesion of glomerular cells, cell movement, cell proliferation of kidney cell lines, cell spreading of kidney cell lines, generation of reactive oxygen species, metabolism of reactive oxygen species, microtubule dynamics, quantity of lipid peroxide, and quantity of superoxide (Fig 8B).

To validate the proteomic analysis findings from the LC-MS/MS of the effects of diabetes on the expression of proteins in the kidney samples, IHC staining for cofilin1 was carried out. The results shown in Figure C in S1 File indicated a decrease in the cofilin1 staining in the kidney sections of the diabetic samples (0.4±0.05-fold) and insulin-treated diabetic samples (0.5±0.06-fold) compared to the control samples.

**Fig 9:**
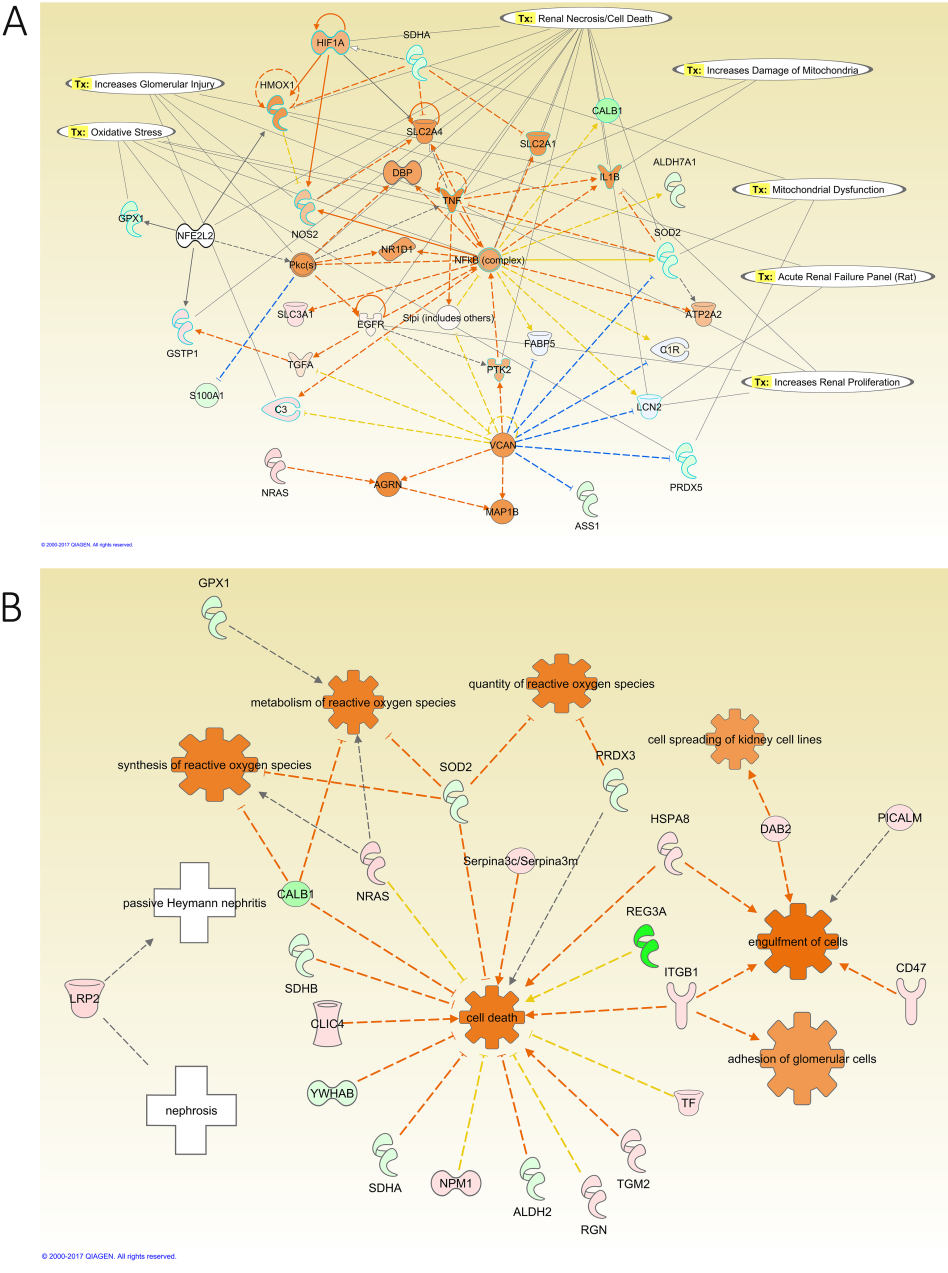
IPA network analysis of the modified proteins in the kidney of insulin-treated diabetic relative to diabetic rats. **A:** The top Toxicity and functions predicted by IPA related to the modified proteins are Increase Damage to Mitochondria, Glomerular Injury, Renal Proliferation, Necrosis/Cell Death, Oxidative Stress, Acute Renal Failure Panel (Rat), and Mitochondrial Dysfunction. The main regulated proteins in this network are NFkB (complex), PRDX5, and SOD2. **B:** Diseases and Biological Functions predicted by IPA related to the differentially expressed proteins in this comparative group. The main biological functions altered in this network are adhesion of glomerular cells and metabolism and synthesis of reactive oxygen species. The diseases related to these proteins are nephrosis, passive Heymann nephritis. The color intensity of the nodes reflects the expression of the proteins. Green nodes are downregulated proteins, red nodes are upregulated proteins, white nodes are IPA predicted proteins with non-consistent activation pattern, and orange nodes are IPA predicted proteins to be activated.

Network analysis of the effect of insulin treatment on the kidney of diabetic rats showed that the upregulation of complement C3 (C3) and ITGB1 are connected to the activation of NFκB and TNF (Fig 9A). Additionally, the upregulation of Gamma-Glutamyltransferase 1 (GGT1), the downregulation of GPX1, peroxiredoxin 5 (PRDX5), and SOD2, and the activation of NFκB and TNF regulated oxidative stress in this group. Moreover, the downregulation of Aconitase 2 (ACO2), oxoglutarate dehydrogenase (OGDH), ATP Synthase, H+ Transporting, Mitochondrial F1 Complex, Beta Polypeptide (ATP5B), SOD2, Succinate Dehydrogenase Complex Flavoprotein Subunit A (SDHA), PRDX5, NADH dehydrogenase [ubiquinone] iron-sulfur protein 2 (NDUFS2), NADH:ubiquinone oxidoreductase core subunit S7 (NDUFS7), and Cytochrome C Oxidase Subunit 4I1 (COX4I1), and the activation of NFκB, and TNF regulated mitochondrial dysfunction in this group (Fig 9B). Furthermore, the modified proteins in the kidney of this group showed activation of adhesion of glomerular cells, cell death, cell spreading of kidney cell lines, engulfment of cells, metabolism of reactive oxygen species, the quantity of reactive oxygen species, and synthesis of reactive oxygen species as Biological Functions.

## Discussion

Discovery proteomic analysis of biological tissues and fluids is increasingly employed in the identification of novel biomarkers. Unlike genomic and transcriptomic approaches for biomarker discovery, proteomics provides significant insights into protein abundance and PTM that modulate protein function and activity [23,24]. In this study, we employed mass spectrometric analysis by using tandem LC-MS/MS to generate a proteomic profile that encompasses information on both protein abundance and post-translational modification in the aorta and kidney of type 1 diabetic rats. In addition, systems biology analysis was employed on modified proteins to identify key proteins that can highlight mechanisms and pathways involved in diabetic vascular and renal biology.

Our data demonstrated that the aorta and kidney responded to the diabetic state differently as evidenced by the proteomic profile of modified proteins expressed in these tissues. Diabetes induced the expression of fibrotic, inflammatory, oxidative and cytoskeleton-related proteins that are interconnected to cellular networks involved in vascular and renal diseases. Insulin treatment of diabetic rats partially reversed the expression profile of the proteins in the aorta and kidney that were significantly modified by the diabetic state, despite normalization of blood glucose levels at study end. This phenomenon is termed “metabolic memory” to specify the continuing persistence of hyperglycemic burden even after glycemic control [25]. Epigenetic modifications of genes caused by the persistent exposure to hyperglycemia may explain some of the enduring detrimental effects of hyperglycemia on causing tissue damage in diabetes [26–29].

It is also conceivable that factors other than hyperglycemia, may have contributed to the expression profile of proteins modified by diabetes. In this regard, our data indicated that the insulin treatment induced the expression of angiotensinogen and ACE in the aorta of diabetic rats, which could result in increased generation of the vasoactive peptide angiotensin II. The generated angiotensin II can act in a paracrine and autocrine manner on vascular cells to mediate a multitude of cellar signaling pathways that results in vascular remodeling [30]. In fact, angiotensin II has been shown to modulate vascular tone and to promote vascular smooth muscle cell proliferation and matrix expansion and contributes to vascular disease in diabetes [31].

Along these lines, analysis of the proteome profile of proteins indicated that diabetes induced the expression levels of matrix proteins such as collagen type VI α6 and type XVIII α1, fibulin 1 and transforming growth factor beta (TGF- β) in the aorta. In this regard,angiotensin II-induced myocardial fibrosis mediated by TGF-β is modulated by fibulin [32]. Moreover, our data indicated that the expression of prohibitin 1 and 2 was reduced in the aorta of diabetic rats, thus increasing the susceptibility to vascular injury. Prohibitin overexpression was associated with inhibition of collagen accumulation and reduction of reactive oxygen species generation in diabetes [33–36]. The downregulation of prohibitin expression in the aorta was associated with the promotion of cell death functions.

Analysis of the proteome profile revealed that diabetes induced the expression of kininogen in both the aorta and kidney and this effect was modulated by hyperglycemia. In addition treatment of diabetic rats with insulin to control their blood glucose levels reversed the expression of kininogen. Upregulation of kininogen was also reported in the urine of T1DM rat model by Caseiro et al. and in the plasma of T1DM patients at risk for renal disease [37,38]. Although, there is paucity of reports that assess the proteomic profile in the aorta of type 1 diabetic rats, Dwinovan et al. employed proteomic analysis to assess the protein abundance in the aorta of type 2 diabetic rats. Among the altered biological pathways in the diabetic group, they have reported that inflammatory and oxidative stress related proteins to be modified by diabetes [39], which is in support of our data in the aorta of type 1 diabetes demonstrating similar alterations in biological processes. In addition our proteomic profiling data in the kidney are consistent with previous reports using type 1 diabetic animal models showing that the expression of calbindin, integrin B1, kininogen 1, haptoglobin, and glutathione peroxidase 1 are altered by the diabetic state [40,41].

Moreover, the effects of diabetes on kininogen expression are in-line with our previous data where we found that the expression and activity of plasma prekallikrein (PK), the serine protease that cleaves kininogen to liberate the vasoactive peptide bradykinin, were elevated in T1DM patients. Furthermore, PK levels were shown to be associated with macroalbuminuria, and carotid intimal medial thickness implicating a role for PK as a risk factor for vascular and renal disease in T1DM patients [42,43]. In addition, targeted deletion of bradykinin receptor 2 in diabetic mice conferred renoprotection against the development of diabetic nephropathy [44]. It is of interest to point here that network analysis of this group of proteins depicted that kininogen and protein kinase A are connected to the activation of angiotensin II type 2, leptin and heme oxygenase 1, thus linking the kallikrein kinin system and renin-angiotensin system to the inflammatory and oxidative stress processes inherent in the diabetic state.

PTMs have been recognized as modulators of target protein resulting in their structural conformational changes that influence their activity, spatial localization and binding to other cellular protein partners [45,46]. Virtually all cellular pathways are regulated by PTMs and their dysregulation has been shown to be related to diseases such as CKD and CVD [47–49]. Our data indicated that diabetes increased the intensities of oxidized, phosphorylated, and acetylated proteins in both the aorta and kidney. This increase in PTMs was independent of protein abundance. Hyperglycemia is recognized as one of the main factors that provoke disproportionate reactive oxygen species production in the vasculature, kidney, and heart [50–52]. Oxidative stress causes modifications of protein, lipids, and DNA and it activates transcription factors that drive inflammation, fibrosis, and cell hypertrophy [26,27,53]. In this regard, our analysis of the proteome profile indicated that diabetes induced the expression of a number of oxidative stress related enzymes involved in hydrogen peroxide, electrophilic, and superoxide detoxification such as glutathione peroxidase 1 and 3, glutathione S-transferase Pi 1, Mu 1, Mu 3, and zeta 1 and monoamine oxidase A, underscoring the oxidative stress condition associated with the exposure of cells to hyperglycemia.

Pathway analysis of our proteome profile indicated that diabetes is associated with activation of inflammatory and oxidative response proteins such as TNF–α, NFκB, leptin, leptin receptors, and p38 MAPK, factors that have been shown to contribute to micro and macrovascular complications of diabetes [54,55]. In addition, our data indicated that diabetes promotes renal cell necrosis through the alteration of the expression of heat shock protein family A (Hsp70) Member 5 (also known as 78 kDa glucose-regulated protein), superoxide dismutase 2, heme oxygenase 1, peroxiredoxin 3, kininogen, peroxisome proliferator-activated receptor gamma (PPARγ) and calbindin 1. The upregulation of calbindin, a vitamin D binding proteins, is suggested to be related to acute renal failure and long-term renal injury panels [56,57]. In addition, our data indicated that prohibitin 1 and 2 are upregulated in the kidney by diabetes and may have a potential role in early stages of diabetic nephropathy [58].

In summary, we have identified a number of proteins that were differentially expressed and post-translationally modified by diabetes in the aorta and kidney. Using informatics analysis of the modified proteome profiles identified key proteins that provided novel insights into biochemical pathways and processes that may be involved in the development of renal and vascular disease in diabetes.

## Supporting information

S1 File Proteome profiling supplementary information. (DOCX)

S2 File Protein group data. (TXT)
