# Supplementary material for: Proteome profiling in the aorta and kidney of type 1 diabetic rats
Source: PLoS One. 2017 Nov 9;12(11):e0187752. doi: 10.1371/journal.pone.0187752 (PMC5679573; doi:10.1371/journal.pone.0187752)
Supplement: S1 File — (DOCX) [file pone.0187752.s001.docx]

**Supporting Methods:**

*Immunohistochemistry of Aorta and Kidney:* Immunohistochemistry (IHC) staining was utilized to assess levels of Transforming Growth Factor Beta (TGFβ) and Cofilin1 expression in aorta and kidney cortex sections, respectively, among the different study groups. Briefly, 5 µm paraffin embedded aortic and kidney sections were prepared using Microtome (Leica Biosystems, Wetzlar, Germany). Slides were deparaffinized and rehydrated through incubations in Xylol and decreasing concentrations of ethanol. Slides were then incubated with antigen retrieval buffer (Citric acid 0.1M, sodium citrate 0.1M, pH 6) for 1 hour at 100ºC. Slides were stained with antibodies against either TGFβ (MAB1032, Chemicon Int., CA, USA) or cofilin1 (ab134963, Abcam, Cambridge, MA, USA) for overnight incubation in cold room, then washed 4 times with PBS for 5 minutes each. TGFβ-stained slides were then incubated with anti-mouse conjugated to Alexa Fluor 647 (Abcam, Cambridge, MA, USA) and cofilin1-stained slides were incubated with anti-rabbit conjugated to FITC (Abcam, Cambridge, MA, USA), for 1 hour at room temperature, then washed 4 times with PBS for 5 minutes each. Nuclei of the tissues were stained with Bisbenzimide (Hoechst 33342 stain, Sigma-Aldrich, Taufkirchen, Germany). Images of tissues were acquired using Laser Scanning Confocal Microscope (Leica Microsystems, Cambridge, UK). Zen 2011 was used to quantify the intensity of the Fluorescence of TGFβ and cofilin1. Fold change of the signal was calculated by rectifying the intensities of TGFβ and cofilin1 relative to nuclei stain relative to control samples.

**Table A: *Comparative list of proteins in the aorta of diabetic rats compared to controls.***

|  | Accession Number | Names | Abbreviation | Fold Change | p value |
| --- | --- | --- | --- | --- | --- |
| 1 | A0A0G2JSJ2 | Cytidine/Uridine Monophosphate Kinase 1 | CMPK1 | 0.47 | 0.001 |
| 2 | A0A0G2JSK5 | Integrin Subunit Beta 1 | ITGB1 | 2.77 | 0.044 |
| 3 | A0A0G2JSS9 | Atlastin-3 | Atl3 | 5.76 | 0.048 |
| 4 | G3V9Q3 | Heterogeneous Nuclear Ribonucleoprotein H | Hnrnph1 | 1.94 | 0.049 |
| 5 | A0A0G2JTL5 | Pyruvate Carboxylase | PC | 0.01 | 0.009 |
| 6 | A0A0G2JTW9 | Hemoglobin Subunit Beta | HBB | 0.33 | 0.034 |
| 7 | F7EPH4 | Pyrophosphatase (Inorganic) 1 | PPA1 | 0.46 | 0.018 |
| 8 | A0A0G2JV31 | X-Prolyl Aminopeptidase 1 | XPNPEP1 | 3.23 | 0.007 |
| 9 | A0A0G2JVH4 | Inner Membrane Mitochondrial Protein | IMMT | 0.20 | 0.039 |
| 10 | F1LUV9 | Neural Cell Adhesion Molecule 1 | Ncam1 | 0.57 | 0.032 |
| 11 | A0A0G2K0Q7 | Myosin Light Chain Kinase | MYLK | 10.44 | 0.046 |
| 12 | A0A0G2K0Z7 | Glycerol-3-Phosphate Dehydrogenase 2 | GPD2 | 0.01 | 0.006 |
| 13 | A0A0G2K167 | Regulator of Microtubule Dynamics Protein 1 | Rmdn1 | 0.09 | 0.010 |
| 14 | A0A0G2K1C0 | ARP3 Actin Related Protein 3 Homolog | ACTR3 | 2.44 | 0.019 |
| 15 | F1LQQ1 | Malic Enzyme 1 | ME1 | 0.20 | 0.017 |
| 16 | A0A0G2K401 | Propionyl-CoA Carboxylase Alpha Subunit | PCCA | 0.02 | 0.008 |
| 17 | A0A0G2K7P7 | Mitochondrial Carrier 2 | MTCH2 | 0.11 | 0.036 |
| 18 | A0A0G2K4M4 | Pro-Epidermal Growth Factor-Like | LOC100910178 | 0.17 | 0.023 |
| 19 | A0A0G2K531 | Glutathione Peroxidase 3 | GPX3 | 2.12 | 0.004 |
| 20 | D3Z8D7 | 40s Ribosomal Protein S26 | LOC100361854 | 0.56 | 0.044 |
| 21 | A0A0G2K7K2 | Apoptosis Inducing Factor Mitochondria Associated 1 | AIFM1 | 0.11 | 0.044 |
| 22 | A0A0G2K8Q1 | Apolipoprotein C-III | Apoc3 | 0.13 | 0.002 |
| 23 | A0A0G2K8Q8 | Ubiquinol-Cytochrome C Reductase, Complex Iii Subunit X | UQCR10 | 0.41 | 0.033 |
| 24 | R9PXU6 | Vinculin | VCL | 2.95 | 0.023 |
| 25 | Q66H18 | Synaptophysin-Like 1 | Sypl1 | 10.00 | 0.001 |
| 26 | A0A0G2KAM3 | Pyruvate Dehydrogenase (Lipoamide) Beta | PDHB | 0.21 | 0.040 |
| 27 | A0A0G2KB63 | Prohibitin 2 | PHB2 | 0.42 | 0.019 |
| 28 | A0A0H2UHM5 | Protein Disulfide Isomerase Family A Member 3 | PDIA3 | 1.64 | 0.022 |
| 29 | A0A0H2UI21 | Carnitine O-Acetyltransferase | CRAT | 0.14 | 0.023 |
| 30 | A0A0U1RRV7 | Rcg61099, Isoform Cra_B | Srsf3 | 6.75 | 0.008 |
| 31 | A0JPK5 | Abhydrolase Domain Containing 5 | ABHD5 | 0.39 | 0.018 |
| 32 | A1L114 | Fibrinogen Alpha Chain | FGA | 0.56 | 0.034 |
| 33 | A1L1M0 | Protein Kinase Camp-Activated Catalytic Subunit Alpha | PRKACA | 1.65 | 0.022 |
| 34 | B2GV33 | Monoamine Oxidase A | MAOA | 2.67 | 0.049 |
| 35 | B2GV73 | Actin Related Protein 2/3 Complex Subunit 3 | ARPC3 | 3.02 | 0.004 |
| 36 | B2RZ24 | Succinate-CoA Ligase ADP-Forming Beta Subunit | SUCLA2 | 0.07 | 0.005 |
| 37 | B2RZA6 | Tbl1x Protein | Tbl1x | 0.55 | 0.011 |
| 38 | F1LSP2 | Acyl-CoA Dehydrogenase Family, Member 10 | Acad10 | 0.14 | 0.027 |
| 39 | B5DEY8 | Sorting Nexin | Snx6 | 1.93 | 0.021 |
| 40 | B5DF65 | Biliverdin Reductase B | BLVRB | 0.42 | 0.044 |
| 41 | B6DYQ7 | Glutathione S-Transferase Pi 1 | GSTP1 | 2.04 | 0.021 |
| 42 | C0KUC5 | Lim Zinc Finger Domain Containing 1 | LIMS1 | 3.27 | 0.037 |
| 43 | D3ZD09 | Cytochrome C Oxidase Subunit 6b1 | COX6B1 | 0.24 | 0.025 |
| 44 | D3ZF13 | NADH:Ubiquinone Oxidoreductase Subunit AB1 | NDUFAB1 | 0.28 | 0.020 |
| 45 | D3ZFQ8 | Cytochrome C1 | CYC1 | 0.31 | 0.048 |
| 46 | D3ZG43 | NADH:Ubiquinone Oxidoreductase Core Subunit S3 | NDUFS3 | 0.08 | 0.030 |
| 47 | D3ZIC4 | Protein Phosphatase 1, Regulatory (Inhibitor) Subunit 12b | Ppp1r12b | 4.20 | 0.007 |
| 48 | D3ZJX5 | Translocase of Inner Mitochondrial Membrane 50 | TIMM50 | 0.20 | 0.006 |
| 49 | D3ZKG1 | Methylmalonyl-CoA Mutase | MUT | 0.09 | 0.006 |
| 50 | D3ZL10 | Collagen Type VI Alpha 6 Chain | COL6A6 | 4.32 | 0.030 |
| 51 | D3ZQ25 | Fibulin 1 | FBLN1 | 4.12 | 0.013 |
| 52 | D3ZS55 | Somatomedin B and Thrombospondin Type 1 Domain Containing | SBSPON | 3.11 | 0.026 |
| 53 | D3ZUX5 | Trans-L-3-Hydroxyproline Dehydratase | L3HYPDH | 0.15 | 0.010 |
| 54 | D3ZV91 | NADH:Ubiquinone Oxidoreductase Subunit B10 | NDUFB10 | 0.31 | 0.046 |
| 55 | D3ZVS2 | L-2-Hydroxyglutarate Dehydrogenase | L2hgdh | 0.25 | 0.042 |
| 56 | D4A0T0 | NADH:Ubiquinone Oxidoreductase Subunit B10 | Ndufb10 | 0.15 | 0.013 |
| 57 | D4A197 | Methylmalonyl-CoA Epimerase | MCEE | 0.10 | 0.038 |
| 58 | D4A2K1 | 4-Hydroxy-2-Oxoglutarate Aldolase 1 | HOGA1 | 0.40 | 0.033 |
| 59 | Q4FZZ4 | Pyruvate Dehydrogenase (Lipoamide) Alpha 1 | PDHA1 | 0.23 | 0.018 |
| 60 | D4A5L9 | Uncharacterized Protein | LOC679794 | 0.21 | 0.002 |
| 61 | G3V6A9 | Microfibril-Associated Glycoprotein 4-Like | LOC102553715 | 2.07 | 0.042 |
| 62 | D4ADF5 | Programmed Cell Death Protein 5-Like | Pdcd5 | 5.60 | 0.011 |
| 63 | F1LNF7 | Isocitrate Dehydrogenase 3 (NAD(+)) Alpha | IDH3A | 0.13 | 0.014 |
| 64 | F1LR02 | Collagen Type XVIII Alpha 1 Chain | COL18A1 | 2.75 | 0.035 |
| 65 | F1LRJ9 | Neural Cell Adhesion Molecule 1 | NCAM1 | 2.04 | 0.037 |
| 66 | F1LTJ5 | Uncharacterized Protein | N/A | 2.23 | 0.019 |
| 67 | Q6P7A4 | Prosaposin | PSAP | 0.36 | 0.026 |
| 68 | G3V624 | Coronin | Coro1c | 19.03 | 0.021 |
| 69 | O54755 | NADH:Ubiquinone Oxidoreductase Subunit V3 | NDUFV3 | 0.06 | 0.014 |
| 70 | G3V734 | 2,4-Dienoyl-CoA Reductase 1 | DECR1 | 0.10 | 0.048 |
| 71 | G3V796 | Acyl-CoA Dehydrogenase, C-4 To C-12 Straight Chain | ACADM | 0.02 | 0.001 |
| 72 | G3V7I0 | Peroxiredoxin 3 | PRDX3 | 0.14 | 0.025 |
| 73 | G3V8R5 | Lipase E, Hormone Sensitive Type | LIPE | 0.09 | 0.021 |
| 74 | G3V928 | LDL Receptor Related Protein 1 | LRP1 | 1.47 | 0.020 |
| 75 | G3V936 | Citrate Synthase | CS | 0.25 | 0.041 |
| 76 | G3V940 | Coronin 1B | CORO1B | 8.55 | 0.007 |
| 77 | G3V9E3 | Caldesmon 1 | Cald1 | 21.46 | 0.046 |
| 78 | G3V9U2 | Acetyl-CoA Acyltransferase 2 | ACAA2 | 0.08 | 0.048 |
| 79 | Q5BJ93 | Enolase 1 | ENO1 | 0.70 | 0.015 |
| 80 | M0R757 | Elongation Factor 1-Alpha | LOC100360413 | 1.95 | 0.017 |
| 81 | M0R7G4 | Apolipoprotein O | Apoo | 0.08 | 0.002 |
| 82 | M0RAM5 | Glutathione Peroxidase 1 | GPX1 | 3.22 | 0.041 |
| 83 | M0RBF1 | Complement C3 | C3 | 0.64 | 0.016 |
| 84 | O35077 | Glycerol-3-Phosphate Dehydrogenase 1 | GPD1 | 0.05 | 0.021 |
| 85 | O35509 | Rab11b, Member Ras Oncogene Family | RAB11B | 1.49 | 0.047 |
| 86 | O35567 | 5-Aminoimidazole-4-Carboxamide Ribonucleotide Formyltransferase/Imp Cyclohydrolase | ATIC | 1.76 | 0.029 |
| 87 | O35796 | Complement C1q Binding Protein | C1QBP | 0.26 | 0.015 |
| 88 | O55096 | Dipeptidyl Peptidase 3 | DPP3 | 1.73 | 0.010 |
| 89 | P04182 | Ornithine Aminotransferase | OAT | 4.90 | 0.050 |
| 90 | P04633 | Uncoupling Protein 1 | UCP1 | 0.00 | 0.004 |
| 91 | P04636 | Malate Dehydrogenase 2 | MDH2 | 0.33 | 0.035 |
| 92 | P04638 | Apolipoprotein A2 | APOA2 | 0.23 | 0.004 |
| 93 | P04639 | Apolipoprotein A1 | APOA1 | 2.29 | 0.032 |
| 94 | P04797 | Glyceraldehyde-3-Phosphate Dehydrogenase | GAPDH | 1.70 | 0.019 |
| 95 | P07340 | ATPase Na+/K+ Transporting Subunit Beta 1 | ATP1B1 | 8.54 | 0.025 |
| 96 | P07379 | Phosphoenolpyruvate Carboxykinase 1 | PCK1 | 0.12 | 0.042 |
| 97 | Q6LDS4 | Superoxide Dismutase 1 | SOD1 | 0.59 | 0.044 |
| 98 | P08461 | Dihydrolipoamide S-Acetyltransferase | DLAT | 0.08 | 0.013 |
| 99 | Q6LCA5 | Protein Kinase cAMP-Dependent Type I Regulatory Subunit Alpha | PRKAR1A | 2.56 | 0.020 |
| 100 | P09606 | Glutamate-Ammonia Ligase | GLUL | 0.24 | 0.027 |
| 101 | P0DN35 | NADH Dehydrogenase [Ubiquinone] 1 Beta Subcomplex Subunit 1 | Ndufb1 | 0.17 | 0.012 |
| 102 | P10888 | Cytochrome C Oxidase Subunit 4I1 | COX4I1 | 0.11 | 0.012 |
| 103 | Q6TXF3 | Diazepam Binding Inhibitor, Acyl-CoA Binding Protein | DBI | 0.57 | 0.024 |
| 104 | P11951 | Cytochrome C Oxidase Subunit Vic | Cox6c | 0.22 | 0.034 |
| 105 | P12007 | Isovaleryl-CoA Dehydrogenase | IVD | 0.02 | 0.002 |
| 106 | P13803 | Electron Transfer Flavoprotein Alpha Subunit | ETFA | 0.11 | 0.039 |
| 107 | P14046 | Murinoglobulin 1 | Mug1 (includes others) | 0.25 | 0.000 |
| 108 | P14408 | Fumarate Hydratase | FH | 0.17 | 0.004 |
| 109 | P14604 | Enoyl-CoA Hydratase, Short Chain 1 | ECHS1 | 0.24 | 0.021 |
| 110 | P15650 | Acyl-CoA Dehydrogenase, Long Chain | ACADL | 0.13 | 0.013 |
| 111 | Q6IMX3 | Acyl-CoA Dehydrogenase, C-2 To C-3 Short Chain | ACADS | 0.20 | 0.027 |
| 112 | R9PXS4 | Phosphatidylinositol Transfer Protein Alpha | PITPNA | 2.50 | 0.003 |
| 113 | P17764 | Acetyl-CoA Acetyltransferase 1 | ACAT1 | 0.16 | 0.016 |
| 114 | P18163 | Acyl-CoA Synthetase Long-Chain Family Member 1 | ACSL1 | 0.10 | 0.024 |
| 115 | P19234 | NADH:Ubiquinone Oxidoreductase Core Subunit V2 | NDUFV2 | 0.15 | 0.022 |
| 116 | P20070 | Cytochrome B5 Reductase 3 | Cyb5r3 | 2.04 | 0.018 |
| 117 | P20788 | Ubiquinol-Cytochrome C Reductase, Rieske Iron-Sulfur Polypeptide 1 | UQCRFS1 | 0.22 | 0.008 |
| 118 | Q6P6T6 | Cathepsin D | CTSD | 0.41 | 0.024 |
| 119 | P97601 | Heat Shock Protein Family E (Hsp10) Member 1 | HSPE1 | 0.36 | 0.025 |
| 120 | P29975 | Aquaporin 1 (Colton Blood Group) | AQP1 | 1.89 | 0.018 |
| 121 | P32198 | Carnitine Palmitoyltransferase 1a | CPT1A | 8.04 | 0.007 |
| 122 | P32551 | Ubiquinol-Cytochrome C Reductase Core Protein II | UQCRC2 | 0.23 | 0.015 |
| 123 | P36201 | Cysteine Rich Protein 2 | Crip2 | 16.44 | 0.006 |
| 124 | P40112 | Proteasome Subunit Beta 3 | PSMB3 | 8.27 | 0.007 |
| 125 | P40307 | Proteasome Subunit Beta 2 | PSMB2 | 4.30 | 0.035 |
| 126 | P50475 | Alanyl-tRNA Synthetase | AARS | 0.31 | 0.020 |
| 127 | P54313 | G Protein Subunit Beta 2 | GNB2 | 1.89 | 0.010 |
| 128 | P62076 | Mitochondrial Import Inner Membrane Translocase Subunit Tim13 | Timm13 | 0.06 | 0.038 |
| 129 | P62142 | Protein Phosphatase 1 Catalytic Subunit Beta | PPP1CB | 7.07 | 0.012 |
| 130 | P62828 | Ran, Member Ras Oncogene Family | RAN | 1.86 | 0.000 |
| 131 | P67779 | Prohibitin | PHB | 0.24 | 0.013 |
| 132 | P68035 | Actin, Alpha, Cardiac Muscle 1 | ACTC1 | 2.12 | 0.049 |
| 133 | P68255 | Tyrosine 3-Monooxygenase/Tryptophan 5-Monooxygenase Activation Protein Theta | YWHAQ | 1.40 | 0.040 |
| 134 | P69897 | Tubulin Beta Class I | TUBB | 2.95 | 0.005 |
| 135 | P85515 | Alpha-Centractin | Actr1a | 3.79 | 0.012 |
| 136 | P85834 | Elongation Factor Tu, Mitochondrial | Tufm | 0.16 | 0.035 |
| 137 | P85973 | Purine Nucleoside Phosphorylase | PNP | 3.39 | 0.009 |
| 138 | Q03336 | Regucalcin | RGN | 0.19 | 0.026 |
| 139 | Q07116 | Sulfite Oxidase | SUOX | 0.06 | 0.000 |
| 140 | Q07936 | Annexin A2 | ANXA2 | 1.80 | 0.042 |
| 141 | Q3MIE4 | Synaptic Vesicle Membrane Protein Vat-1 Homolog | Vat1 | 1.35 | 0.013 |
| 142 | Q499N5 | Acyl-CoA Synthetase Family Member 2, Mitochondrial | Acsf2 | 0.03 | 0.023 |
| 143 | Q4QRB4 | Tubulin Beta 3 Class Iii | TUBB3 | 0.13 | 0.002 |
| 144 | Q4V8N0 | Lipocalin 7, Isoform Cra_A | Tinagl1 | 2.18 | 0.047 |
| 145 | Q561S0 | NADH:Ubiquinone Oxidoreductase Subunit A10 | NDUFA10 | 0.04 | 0.001 |
| 146 | Q56R17 | Karyopherin Subunit Alpha 4 | KPNA4 | 0.36 | 0.048 |
| 147 | Q5BJZ3 | Nicotinamide Nucleotide Transhydrogenase | NNT | 15.04 | 0.034 |
| 148 | Q5EB77 | Rab18, Member Ras Oncogene Family | RAB18 | 0.68 | 0.041 |
| 149 | Q5I0E7 | Transmembrane Emp24 Domain-Containing Protein 9 | Tmed9 | 12.35 | 0.015 |
| 150 | Q5M7U6 | ARP2 Actin Related Protein 2 Homolog | ACTR2 | 17.53 | 0.000 |
| 151 | Q5M9H2 | Acyl-CoA Dehydrogenase, Very Long Chain | ACADVL | 0.22 | 0.024 |
| 152 | Q5M9I5 | Ubiquinol-Cytochrome C Reductase Hinge Protein Like | UQCRHL | 0.20 | 0.014 |
| 153 | Q5PQU1 | Kininogen 1 | Kng1/Kng1l1 | 2.91 | 0.004 |
| 154 | Q5PQZ9 | NADH:Ubiquinone Oxidoreductase Subunit C2 | NDUFC2 | 0.06 | 0.026 |
| 155 | Q5XI77 | Annexin | Anxa11 | 2.26 | 0.014 |
| 156 | Q5XI78 | Oxoglutarate Dehydrogenase | OGDH | 0.06 | 0.024 |
| 157 | Q5XIF3 | NADH:Ubiquinone Oxidoreductase Subunit S4 | NDUFS4 | 0.14 | 0.012 |
| 158 | Q5XIT9 | Methylcrotonoyl-CoA Carboxylase 2 | MCCC2 | 0.05 | 0.005 |
| 159 | Q60587 | Hydroxyacyl-CoA Dehydrogenase/3-Ketoacyl-CoA Thiolase/Enoyl-CoA Hydratase (Trifunctional Protein), Beta Subunit | HADHB | 0.08 | 0.013 |
| 160 | Q62952 | Dihydropyrimidinase Like 3 | DPYSL3 | 3.89 | 0.015 |
| 161 | Q62969 | Prostaglandin I2 Synthase | PTGIS | 3.75 | 0.027 |
| 162 | Q63704 | Carnitine Palmitoyltransferase 1b | CPT1B | 0.04 | 0.011 |
| 163 | Q641Y2 | NADH:Ubiquinone Oxidoreductase Core Subunit S2 | NDUFS2 | 0.07 | 0.044 |
| 164 | Q64428 | Hydroxyacyl-CoA Dehydrogenase/3-Ketoacyl-CoA Thiolase/Enoyl-CoA Hydratase (Trifunctional Protein), Alpha Subunit | HADHA | 0.18 | 0.019 |
| 165 | Q64542 | ATPase Plasma Membrane Ca2+ Transporting 4 | ATP2B4 | 6.92 | 0.042 |
| 166 | Q66HF1 | NADH:Ubiquinone Oxidoreductase Core Subunit S1 | NDUFS1 | 0.16 | 0.012 |
| 167 | Q68FT1 | Coenzyme Q9 | COQ9 | 0.03 | 0.024 |
| 168 | Q68FU3 | Electron Transfer Flavoprotein Subunit Beta | Etfb | 0.08 | 0.011 |
| 169 | Q68FY0 | Ubiquinol-Cytochrome C Reductase Core Protein I | UQCRC1 | 0.10 | 0.008 |
| 170 | Q6MG61 | Chloride Intracellular Channel 1 | CLIC1 | 1.48 | 0.036 |
| 171 | Q6P6R2 | Dihydrolipoamide Dehydrogenase | DLD | 0.15 | 0.022 |
| 172 | Q9WVJ6 | Transglutaminase 2 | TGM2 | 2.64 | 0.014 |
| 173 | Q6P7P5 | Basic Leucine Zipper and W2 Domain-Containing Protein 1 | Bzw1 | 0.23 | 0.002 |
| 174 | Q6P9V1 | CD81 Molecule | CD81 | 3.49 | 0.044 |
| 175 | Q6P9V6 | Proteasome Subunit Alpha 5 | PSMA5 | 1.44 | 0.006 |
| 176 | Q6P9V9 | Tubulin Alpha-1b Chain | Tuba1b | 2.42 | 0.009 |
| 177 | Q6TXG7 | Serine Hydroxymethyltransferase 1 | SHMT1 | 0.01 | 0.001 |
| 178 | Q794E4 | Heterogeneous Nuclear Ribonucleoprotein F | HNRNPF | 1.28 | 0.023 |
| 179 | Q91Y81 | Septin 2 | SEPT2 | 4.37 | 0.018 |
| 180 | Q920L2 | Succinate Dehydrogenase Complex Flavoprotein Subunit A | SDHA | 0.14 | 0.013 |
| 181 | Q924S5 | Lon Peptidase 1, Mitochondrial | LONP1 | 0.20 | 0.019 |
| 182 | Q99PD6 | Transforming Growth Factor Beta 1 Induced Transcript 1 | TGFB1I1 | 9.85 | 0.046 |
| 183 | Q9ER34 | Aconitase 2 | ACO2 | 0.14 | 0.025 |
| 184 | Q9ESN0 | Family with Sequence Similarity 129 Member A | FAM129A | 5.12 | 0.018 |
| 185 | Q9JJ54 | Heterogeneous Nuclear Ribonucleoprotein D | HNRNPD | 2.43 | 0.038 |
| 186 | Q9JLZ1 | Glutaredoxin 3 | GLRX3 | 0.28 | 0.016 |
| 187 | Q9Z0V5 | Peroxiredoxin 4 | PRDX4 | 0.70 | 0.014 |
| 188 | Q9Z1B2 | Glutathione S-Transferase Mu 3 | GSTM3 | 16.78 | 0.039 |

**Table B: *Comparative list of proteins in the kidney of diabetic rats compared to controls.***

|  | Accession Number | Name | Abbreviation | Fold Change | p value |
| --- | --- | --- | --- | --- | --- |
| 1 | A0A0A0MXW3 | H2A Histone Family Member Z | H2AFZ | 0.76 | 0.021 |
| 2 | A0A0A0MXW9 | RNA-Binding Motif Protein, X-Linked-Like-1 | Rbmxl1 | 0.78 | 0.014 |
| 3 | Q95938 | Cytochrome C Oxidase Subunit I (Mitochondrion) | COX1 | 1.21 | 0.047 |
| 4 | A0A0G2JSI0 | Flavin Containing Monooxygenase 3 | FMO3 | 0.61 | 0.049 |
| 5 | A0A0G2JSK5 | Integrin Subunit Beta 1 | ITGB1 | 0.74 | 0.009 |
| 6 | A0A0G2JSV6 | Globin C2 | Hba-a2 | 0.63 | 0.008 |
| 7 | A0A0G2JTL5 | Pyruvate Carboxylase | PC | 1.82 | 0.004 |
| 8 | A0A0G2JTW9 | Hemoglobin Subunit Beta | HBB | 0.49 | 0.028 |
| 9 | D4A6E3 | Murinoglobulin-1 Precursor | Mug1 | 0.24 | 0.011 |
| 10 | A0A0G2JV65 | Tyrosine 3-Monooxygenase/Tryptophan 5-Monooxygenase Activation Protein Zeta | YWHAZ | 0.86 | 0.024 |
| 11 | A0A0G2JVH4 | Inner Membrane Mitochondrial Protein | IMMT | 1.16 | 0.049 |
| 12 | A0A0G2JVL6 | NADH:Ubiquinone Oxidoreductase Subunit A8 | NDUFA8 | 1.66 | 0.028 |
| 13 | D3ZFD0 | Myosin XVIIIA | Myo18a | 1.32 | 0.036 |
| 14 | A0A0G2K4D0 | Ankyrin 3 | ANK3 | 0.79 | 0.003 |
| 15 | A0A0G2K0Q8 | Alkaline Phosphatase, Liver/Bone/Kidney | ALPL | 2.11 | 0.016 |
| 16 | A0A0G2K0X8 | Alkaline Phosphatase | Alpl | 1.37 | 0.039 |
| 17 | Q6PAH0 | Apolipoprotein E | APOE | 0.45 | 0.039 |
| 18 | A0A0G2K277 | Enoyl-Coenzyme A Delta Isomerase 3 | Eci3 | 1.20 | 0.002 |
| 19 | A0A0G2K401 | Propionyl-CoA Carboxylase Alpha Subunit | PCCA | 1.25 | 0.031 |
| 20 | A0A0G2K531 | Glutathione Peroxidase 3 | GPX3 | 0.77 | 0.012 |
| 21 | A0A0U1RRQ2 | Actin Related Protein 2/3 Complex Subunit 5 | ARPC5 | 0.73 | 0.005 |
| 22 | G3V9G4 | ATP Citrate Lyase | ACLY | 0.70 | 0.032 |
| 23 | D3ZCF5 | SMAD Specific E3 Ubiquitin Protein Ligase 1 | SMURF1 | 0.23 | 0.003 |
| 24 | A0A0G2K6H2 | Glutathione S-Transferase Zeta 1 | GSTZ1 | 1.86 | 0.005 |
| 25 | D3Z8D7 | Ribosomal Protein S26 | Rps26 | 0.79 | 0.013 |
| 26 | A0A1B0GWP1 | ELOVL Fatty Acid Elongase 1 | ELOVL1 | 0.71 | 0.002 |
| 27 | A0A0G2K8Q8 | Ubiquinol-Cytochrome C Reductase, Complex III Subunit X | UQCR10 | 1.20 | 0.017 |
| 28 | G3V6S0 | Spectrin Beta, Non-Erythrocytic 1 | SPTBN1 | 0.79 | 0.000 |
| 29 | Q80YG1 | Phosphoserine Aminotransferase 1 | PSAT1 | 0.67 | 0.025 |
| 30 | A0A0G2K9J2 | ATPase H+ Transporting V1 Subunit H | ATP6V1H | 1.59 | 0.005 |
| 31 | A0A0G2K9W7 | LDL Receptor Related Protein 2 | LRP2 | 0.45 | 0.019 |
| 32 | A0A0G2KAM3 | Pyruvate Dehydrogenase (Lipoamide) Beta | PDHB | 1.18 | 0.032 |
| 33 | Q7TNX2 | Peroxisomal Membrane Protein 2 | Pxmp2 | 2.42 | 0.039 |
| 34 | A0A0G2KB63 | Prohibitin 2 | PHB2 | 1.15 | 0.024 |
| 35 | A0A0G2QC04 | Plastin 1 | Pls1 | 0.72 | 0.026 |
| 36 | A0A0H2UHE1 | Succinate-CoA Ligase Alpha Subunit | SUCLG1 | 1.36 | 0.023 |
| 37 | A0A0H2UHE4 | Regenerating Family Member 3 Alpha | REG3A | 10.17 | 0.023 |
| 38 | A0A0H2UHM3 | Haptoglobin | HP | 0.67 | 0.027 |
| 39 | Q7TP86 | Uncharacterized Protein LOC502176 | LOC502176 | 0.78 | 0.032 |
| 40 | A2VCW9 | Aminoadipate-Semialdehyde Synthase | AASS | 2.16 | 0.037 |
| 41 | A4PB92 | Glycine N-Acyltransferase | Glyat | 1.34 | 0.037 |
| 42 | B0BMW2 | Hydroxysteroid 17-Beta Dehydrogenase 10 | HSD17B10 | 1.51 | 0.008 |
| 43 | B0BNG1 | Proline Dehydrogenase 2 | PRODH2 | 1.46 | 0.016 |
| 44 | B1H216 | Hemoglobin, Alpha 1 | Hba1/Hba2 | 0.53 | 0.046 |
| 45 | Q5EBA9 | Succinate-CoA Ligase GDP-Forming Beta Subunit | SUCLG2 | 1.32 | 0.009 |
| 46 | B2GUV5 | Atpase H+ Transporting V1 Subunit G1 | ATP6V1G1 | 0.81 | 0.008 |
| 47 | B2GVB1 | S100 Calcium Binding Protein A6 | S100A6 | 1.96 | 0.000 |
| 48 | B2RYR8 | Ribosomal Protein S8 | RPS8 | 0.72 | 0.003 |
| 49 | B2RYU2 | 60S Ribosomal Protein L12 | Rpl12 | 0.77 | 0.028 |
| 50 | B2RYW3 | NADH:Ubiquinone Oxidoreductase Subunit B9 | NDUFB9 | 1.36 | 0.041 |
| 51 | B2RYW9 | Fumarylacetoacetate Hydrolase Domain-Containing Protein 2 | Fahd2a | 1.74 | 0.013 |
| 52 | B2RZ24 | Succinate-CoA Ligase ADP-Forming Beta Subunit | SUCLA2 | 1.27 | 0.031 |
| 53 | B4F768 | Aldehyde Dehydrogenase 4 Family Member A1 | ALDH4A1 | 1.88 | 0.021 |
| 54 | B5DER3 | Isoamyl Acetate-Hydrolyzing Esterase 1 Homolog | Iah1 | 0.82 | 0.045 |
| 55 | D3ZCH6 | Ribonuclease T2 Precursor | Rnaset2 | 0.74 | 0.018 |
| 56 | B5DFA0 | Villin-1 | Vil1 | 0.75 | 0.019 |
| 57 | B6DYQ2 | Glutathione S-Transferase Mu 1 | GSTM1 | 1.55 | 0.039 |
| 58 | D3Z8F1 | Villin-Like Protein | Vill | 0.64 | 0.010 |
| 59 | D3Z8M8 | TSC22 Domain Family Member 1 | TSC22D1 | 0.76 | 0.030 |
| 60 | M0R6N2 | MOCO Sulphurase C-Terminal Domain Containing 2 | Mosc2 | 1.55 | 0.005 |
| 61 | D4A3B0 | Talin 2 | TLN2 | 0.66 | 0.031 |
| 62 | D3ZFY8 | Ubiquitin-Conjugating Enzyme E2 Variant 1 | Ube2v1 | 0.76 | 0.042 |
| 63 | Q6PDW1 | Ribosomal Protein S12 | RPS12 | 0.86 | 0.041 |
| 64 | D3ZPL5 | 60S Ribosomal Protein L7a | RGD1562953 | 0.74 | 0.035 |
| 65 | D3ZRJ6 | Cell Surface A33 Antigen Precursor | Gpa33 | 1.39 | 0.028 |
| 66 | D3ZSL2 | Uncharacterized Protein LOC685045 | Abracl | 0.15 | 0.047 |
| 67 | D3ZT90 | Glutaryl-CoA Dehydrogenase | GCDH | 1.39 | 0.001 |
| 68 | D3ZTX4 | Maltase-Glucoamylase | Mgam | 0.28 | 0.001 |
| 69 | D3ZXK4 | Abhydrolase Domain Containing 11 | Abhd11 | 0.39 | 0.025 |
| 70 | D3ZXY4 | Aldehyde Dehydrogenase 8 Family Member A1 | ALDH8A1 | 0.32 | 0.000 |
| 71 | D3ZZN3 | Acyl-CoA Synthetase Short-Chain Family Member 1 | ACSS1 | 1.59 | 0.012 |
| 72 | D4A5I9 | Myosin VI | MYO6 | 0.82 | 0.004 |
| 73 | D4A8H3 | Ubiquitin-Like Modifier-Activating Enzyme 6 | Uba6 | 0.56 | 0.007 |
| 74 | D4AB01 | Histidine Triad Nucleotide Binding Protein 2 | Hint2 | 1.39 | 0.036 |
| 75 | F1LMP9 | DAB2, Clathrin Adaptor Protein | DAB2 | 0.59 | 0.012 |
| 76 | F1LMV6 | Desmoplakin | DSP | 0.67 | 0.006 |
| 77 | F1LP30 | Methylcrotonoyl-CoA Carboxylase 1 | MCCC1 | 1.53 | 0.004 |
| 78 | F1LPD6 | Acetyl-CoA Acyltransferase 1 | ACAA1 | 1.52 | 0.006 |
| 79 | F1LRJ9 | Selenium-Binding Protein 1 | Selenbp1 | 1.65 | 0.012 |
| 80 | F1LWL8 | Sodium-Coupled Monocarboxylate Transporter 2 | Slc5a12 | 1.32 | 0.008 |
| 81 | Q8CHN5 | Epididymal Secretory Protein E1 Precursor | Npc2 | 0.71 | 0.019 |
| 82 | G3V6D3 | ATP Synthase, H+ Transporting, Mitochondrial F1 Complex, Beta Polypeptide | ATP5B | 1.25 | 0.002 |
| 83 | G3V6H5 | Solute Carrier Family 25 Member 11 | SLC25A11 | 1.28 | 0.016 |
| 84 | G3V6P2 | Dihydrolipoamide S-Succinyltransferase | DLST | 1.22 | 0.046 |
| 85 | Q5EBA4 | 4-Nitrophenylphosphatase | Nipsnap1 | 1.42 | 0.014 |
| 86 | G3V7I0 | Peroxiredoxin 3 | PRDX3 | 1.36 | 0.001 |
| 87 | G3V7T5 | Xylulose Kinase | Xylb | 1.21 | 0.028 |
| 88 | G3V826 | Transketolase | TKT | 3.07 | 0.014 |
| 89 | G3V8A5 | Maternal Embryonic Message 3 | Vps35 | 0.69 | 0.006 |
| 90 | G3V8D6 | Tripartite Motif Containing 3 | TRIM3 | 0.45 | 0.047 |
| 91 | Q6P784 | Branched Chain Amino Acid Transaminase 2 | BCAT2 | 1.31 | 0.050 |
| 92 | I6L9G6 | TAR DNA Binding Protein | Tardbp | 0.78 | 0.028 |
| 93 | Q5BJ93 | Enolase 1 | ENO1 | 0.77 | 0.011 |
| 94 | M0R7P0 | Protein LOC688784 | LOC688784 | 0.91 | 0.039 |
| 95 | M0RAM5 | Glutathione Peroxidase 1 | GPX1 | 1.49 | 0.047 |
| 96 | M0RBF1 | Complement C3 | C3 | 0.50 | 0.029 |
| 97 | M0RCY2 | Similar to Ribosomal Protein S13 | LOC683961 | 0.67 | 0.047 |
| 98 | M0RDH0 | Glycine N-Methyltransferase | Gnmt | 1.35 | 0.006 |
| 99 | O35244 | Peroxiredoxin 6 | PRDX6 | 1.67 | 0.047 |
| 100 | P00507 | Glutamic-Oxaloacetic Transaminase 2 | GOT2 | 1.35 | 0.006 |
| 101 | P02091 | Hemoglobin Subunit Beta-1 | Hbb | 0.62 | 0.031 |
| 102 | P02680 | Fibrinogen Gamma Chain | FGG | 0.77 | 0.018 |
| 103 | P02761 | Alpha-2u-Globulin (L Type) Precursor | Mup5 | 0.35 | 0.005 |
| 104 | P02770 | Albumin | ALB | 0.64 | 0.004 |
| 105 | P04639 | Apolipoprotein A1 | APOA1 | 2.13 | 0.005 |
| 106 | P04797 | Glyceraldehyde-3-Phosphate Dehydrogenase | GAPDH | 1.22 | 0.026 |
| 107 | Q45QN0 | G Protein Subunit Alpha I2 | GNAI2 | 0.71 | 0.022 |
| 108 | P05065 | Aldolase, Fructose-Bisphosphate A | ALDOA | 0.85 | 0.036 |
| 109 | P06761 | Heat Shock Protein Family A (Hsp70) Member 5 | HSPA5 | 0.90 | 0.023 |
| 110 | P07171 | Calbindin 1 | CALB1 | 1.55 | 0.047 |
| 111 | P07483 | Fatty Acid Binding Protein 3 | FABP3 | 0.77 | 0.012 |
| 112 | P07687 | Epoxide Hydrolase 1 | EPHX1 | 2.35 | 0.002 |
| 113 | P07895 | Superoxide Dismutase 2 | SOD2 | 1.24 | 0.018 |
| 114 | P10860 | Glutamate Dehydrogenase 1 | GLUD1 | 1.22 | 0.010 |
| 115 | P10888 | Cytochrome C Oxidase Subunit 4I1 | COX4I1 | 1.26 | 0.018 |
| 116 | P12346 | Transferrin | TF | 0.63 | 0.002 |
| 117 | P12785 | Fatty Acid Synthase | FASN | 0.43 | 0.036 |
| 118 | P13221 | Glutamic-Oxaloacetic Transaminase 1 | GOT1 | 1.32 | 0.024 |
| 119 | P13803 | Electron Transfer Flavoprotein Alpha Subunit | ETFA | 1.34 | 0.013 |
| 120 | P14046 | Murinoglobulin 1 | Mug1 (includes others) | 0.16 | 0.000 |
| 121 | P14408 | Fumarate Hydratase | FH | 1.22 | 0.023 |
| 122 | P14562 | Lysosomal Associated Membrane Protein 1 | LAMP1 | 0.57 | 0.050 |
| 123 | Q6IMX3 | Acyl-CoA Dehydrogenase, C-2 To C-3 Short Chain | ACADS | 1.38 | 0.000 |
| 124 | P15999 | ATP Synthase, H+ Transporting, Mitochondrial F1 Complex, Alpha Subunit 1, Cardiac Muscle | ATP5A1 | 1.18 | 0.012 |
| 125 | P17764 | Acetyl-CoA Acetyltransferase 1 | ACAT1 | 1.19 | 0.041 |
| 126 | P18163 | Acyl-CoA Synthetase Long-Chain Family Member 1 | ACSL1 | 1.70 | 0.020 |
| 127 | P19112 | Fructose-Bisphosphatase 1 | FBP1 | 0.41 | 0.000 |
| 128 | P19804 | NME/NM23 Nucleoside Diphosphate Kinase 2 | NME2 | 1.06 | 0.021 |
| 129 | P20059 | Hemopexin | HPX | 0.75 | 0.017 |
| 130 | P21913 | Succinate Dehydrogenase Complex Iron Sulfur Subunit B | SDHB | 1.26 | 0.003 |
| 131 | Q68G41 | Enoyl-CoA Delta Isomerase 1 | Eci1 | 2.01 | 0.021 |
| 132 | P97601 | Heat Shock Protein Family E (Hsp10) Member 1 | HSPE1 | 1.26 | 0.017 |
| 133 | P29266 | 3-Hydroxyisobutyrate Dehydrogenase | HIBADH | 1.71 | 0.004 |
| 134 | P31399 | ATP Synthase, H+ Transporting, Mitochondrial Fo Complex Subunit D | ATP5H | 1.32 | 0.013 |
| 135 | P32551 | Ubiquinol-Cytochrome C Reductase Core Protein II | UQCRC2 | 1.14 | 0.044 |
| 136 | P32755 | 4-Hydroxyphenylpyruvate Dioxygenase | HPD | 1.57 | 0.004 |
| 137 | P42123 | Lactate Dehydrogenase B | LDHB | 1.37 | 0.010 |
| 138 | P43428 | Glucose-6-Phosphatase Catalytic Subunit | G6PC | 1.54 | 0.012 |
| 139 | P45592 | Cofilin 1 | CFL1 | 0.72 | 0.002 |
| 140 | P51635 | Aldo-Keto Reductase Family 1 Member A1 | AKR1A1 | 0.95 | 0.017 |
| 141 | P52631 | Signal Transducer and Activator of Transcription 3 | STAT3 | 0.77 | 0.010 |
| 142 | P53987 | Solute Carrier Family 16 Member 1 | SLC16A1 | 1.51 | 0.000 |
| 143 | P54313 | G Protein Subunit Beta 2 | GNB2 | 0.85 | 0.037 |
| 144 | P55053 | Fatty Acid Binding Protein 5 | FABP5 | 0.40 | 0.011 |
| 145 | P56574 | Isocitrate Dehydrogenase (NADP(+)) 2, Mitochondrial | IDH2 | 1.16 | 0.023 |
| 146 | Q5D059 | Heterogeneous Nuclear Ribonucleoprotein K | HNRNPK | 0.88 | 0.014 |
| 147 | P62161 | Calmodulin | Calm1 | 0.84 | 0.041 |
| 148 | P62260 | Tyrosine 3-Monooxygenase/Tryptophan 5-Monooxygenase Activation Protein Epsilon | YWHAE | 0.89 | 0.004 |
| 149 | P62282 | Ribosomal Protein S11 | RPS11 | 0.78 | 0.047 |
| 150 | P62804 | Histone H4 Osteogenic Growth Peptide | Hist2h4 | 0.74 | 0.007 |
| 151 | P62832 | Ribosomal Protein L23 | RPL23 | 0.82 | 0.008 |
| 152 | P63259 | Actin Gamma 1 | ACTG1 | 0.86 | 0.020 |
| 153 | P67779 | Prohibitin | PHB | 1.29 | 0.016 |
| 154 | P68182 | Protein Kinase cAMP-Activated Catalytic Subunit Beta | PRKACB | 0.37 | 0.032 |
| 155 | P68255 | Tyrosine 3-Monooxygenase/Tryptophan 5-Monooxygenase Activation Protein Theta | YWHAQ | 0.84 | 0.012 |
| 156 | P68511 | Tyrosine 3-Monooxygenase/Tryptophan 5-Monooxygenase Activation Protein Eta | YWHAH | 0.56 | 0.029 |
| 157 | P69897 | Tubulin Beta Class I | TUBB | 0.85 | 0.014 |
| 158 | P70473 | Alpha-Methylacyl-CoA Racemase | AMACR | 2.24 | 0.000 |
| 159 | P85968 | Phosphogluconate Dehydrogenase | PGD | 0.72 | 0.036 |
| 160 | Q66HP8 | Solute Carrier Family 25 Member 20 | SLC25A20 | 1.34 | 0.014 |
| 161 | Q66HN6 | Solute Carrier Family 27 Member 2 | SLC27A2 | 1.52 | 0.017 |
| 162 | P97532 | Mercaptopyruvate Sulfurtransferase | MPST | 1.16 | 0.023 |
| 163 | P97584 | Prostaglandin Reductase 1 | PTGR1 | 0.21 | 0.001 |
| 164 | Q03336 | Regucalcin | RGN | 0.47 | 0.034 |
| 165 | Q06647 | ATP Synthase, H+ Transporting, Mitochondrial F1 Complex, O Subunit | ATP5O | 1.17 | 0.025 |
| 166 | Q0D2L3 | Agmatinase, Mitochondrial | Agmat | 1.34 | 0.012 |
| 167 | Q3KR94 | Vitronectin | VTN | 0.52 | 0.006 |
| 168 | Q4KM66 | Immunoglobulin Kappa Constant | Igkc | 1.18 | 0.050 |
| 169 | Q6PPF3 | Harmonin | Ush1c | 0.40 | 0.028 |
| 170 | Q4QQW3 | Alcohol Dehydrogenase, Iron Containing 1 | ADHFE1 | 3.36 | 0.000 |
| 171 | Q4V8F6 | Poly(Rc)-Binding Protein 2 | Pcbp2 | 0.56 | 0.013 |
| 172 | Q562C4 | Methyltransferase-Like Protein 7B | Mettl7b | 1.47 | 0.011 |
| 173 | Q5BJZ3 | Nicotinamide Nucleotide Transhydrogenase | NNT | 1.58 | 0.010 |
| 174 | Q5HZA9 | Transmembrane Protein 126A | Tmem126a | 1.36 | 0.016 |
| 175 | Q5M9H2 | Acyl-CoA Dehydrogenase, Very Long Chain | ACADVL | 1.33 | 0.004 |
| 176 | Q5PPP1 | Clathrin Light Chain A | CLTA | 0.24 | 0.015 |
| 177 | Q5PQU1 | Kininogen 1 | Kng1/Kng1l1 | 4.61 | 0.012 |
| 178 | Q5RJR2 | Twinfilin Actin Binding Protein 1 | TWF1 | 0.18 | 0.049 |
| 179 | Q5RKI0 | WD Repeat-Containing Protein 1 | Wdr1 | 0.73 | 0.040 |
| 180 | Q5U2P9 | Monocarboxylate Transporter 5 | Slc16a4 | 0.83 | 0.049 |
| 181 | Q5XFX0 | Transgelin-2 | Tagln2 | 0.80 | 0.012 |
| 182 | Q5XI34 | Protein Phosphatase 2 Scaffold Subunit Aalpha | PPP2R1A | 0.72 | 0.010 |
| 183 | Q5XI78 | Oxoglutarate Dehydrogenase | OGDH | 1.26 | 0.014 |
| 184 | Q5XI85 | Aminomethyltransferase | AMT | 1.54 | 0.016 |
| 185 | Q5XIC0 | Enoyl-CoA Delta Isomerase 2 | ECI2 | 1.74 | 0.048 |
| 186 | Q5XIH3 | NADH:Ubiquinone Oxidoreductase Core Subunit V1 | NDUFV1 | 1.17 | 0.042 |
| 187 | Q62651 | Enoyl-CoA Hydratase 1 | ECH1 | 1.94 | 0.007 |
| 188 | Q63041 | Alpha-1-Macroglobulin Alpha-1-Macroglobulin 45 kDa Subunit | Pzp | 0.46 | 0.031 |
| 189 | Q63342 | Dimethylglycine Dehydrogenase | DMGDH | 1.45 | 0.002 |
| 190 | Q63530 | Phosphotriesterase-Related Protein | Pter | 0.73 | 0.000 |
| 191 | Q63768 | CRK Proto-Oncogene, Adaptor Protein | CRK | 0.76 | 0.036 |
| 192 | Q63910 | Hemoglobin Subunit Alpha 2 | HBA1/HBA2 | 0.37 | 0.009 |
| 193 | Q64057 | Aldehyde Dehydrogenase 7 Family Member A1 | ALDH7A1 | 1.23 | 0.011 |
| 194 | Q641Y0 | Dolichyl-Diphosphooligosaccharide--Protein Glycosyltransferase 48 kDa Subunit Precursor | Ddost | 0.75 | 0.002 |
| 195 | Q641Y2 | NADH:Ubiquinone Oxidoreductase Core Subunit S2 | NDUFS2 | 1.78 | 0.001 |
| 196 | Q642E6 | Tripeptidyl Peptidase 1 | TPP1 | 1.14 | 0.042 |
| 197 | Q66HF3 | Electron Transfer Flavoprotein-Ubiquinone Oxidoreductase | Etfdh | 1.48 | 0.047 |
| 198 | Q66HG3 | Beta-Ala-His Dipeptidase | Cndp1 | 0.41 | 0.038 |
| 199 | Q68FS4 | Cytosol Aminopeptidase | Lap3 | 1.46 | 0.009 |
| 200 | Q68FU3 | Electron Transfer Flavoprotein Subunit Beta | Etfb | 1.26 | 0.007 |
| 201 | Q68FZ8 | Propionyl-CoA Carboxylase Beta Subunit | PCCB | 1.50 | 0.024 |
| 202 | Q68G31 | Enoyl-CoA Delta Isomerase 1 | ECI1 | 1.49 | 0.006 |
| 203 | Q6JE36 | Protein NDRG1 | Ndrg1 | 1.86 | 0.002 |
| 204 | Q6P6R2 | Dihydrolipoamide Dehydrogenase | DLD | 1.32 | 0.009 |
| 205 | Q6P7P5 | Basic Leucine Zipper and W2 Domain-Containing Protein 1 | Bzw1 | 0.67 | 0.042 |
| 206 | Q6P9V6 | Proteasome Subunit Alpha 5 | PSMA5 | 0.81 | 0.035 |
| 207 | Q812B0 | Succinate Dehydrogenase [Ubiquinone] Cytochrome B Small Subunit | Lrrp1 | 1.18 | 0.019 |
| 208 | Q7TP38 | Proteasome Activator Subunit 3 | PSME3 | 1.89 | 0.046 |
| 209 | Q80ZA3 | Serpin Family F Member 1 | SERPINF1 | 0.60 | 0.003 |
| 210 | Q8CFN2 | Cell Division Cycle 42 | Cdc42 | 0.89 | 0.040 |
| 211 | Q9JJI4 | Crystallin Mu | CRYM | 0.20 | 0.002 |
| 212 | Q8VI04 | Asparaginase Like 1 | ASRGL1 | 0.70 | 0.018 |
| 213 | Q91ZW6 | Trimethyllysine Hydroxylase, Epsilon | TMLHE | 2.78 | 0.002 |
| 214 | Q920A6 | Serine Carboxypeptidase 1 | SCPEP1 | 0.64 | 0.036 |
| 215 | Q920L2 | Succinate Dehydrogenase Complex Flavoprotein Subunit A | SDHA | 1.22 | 0.005 |
| 216 | Q923Z2 | Tropomyosin 1, Alpha | Tpm1 | 0.82 | 0.002 |
| 217 | Q9EPB1 | Dipeptidyl Peptidase 7 | DPP7 | 0.61 | 0.001 |
| 218 | Q9ER34 | Aconitase 2 | ACO2 | 1.42 | 0.007 |
| 219 | Q9JJ54 | Heterogeneous Nuclear Ribonucleoprotein D | HNRNPD | 0.84 | 0.037 |
| 220 | Q9JJW3 | Up-Regulated During Skeletal Muscle Growth Protein 5 | Usmg5 | 1.11 | 0.007 |
| 221 | Q9QYU4 | Thiomorpholine-Carboxylate Dehydrogenase | Crym | 1.81 | 0.012 |
| 222 | Q9Z2L0 | Voltage Dependent Anion Channel 1 | VDAC1 | 1.18 | 0.006 |
| 223 | Q9Z2Y0 | Glycine N-Acyltransferase-Like Protein Keg1 | Keg1 | 1.31 | 0.016 |

**Table C: *Common proteins between aorta and kidney in diabetic vs. control.***

| Accession Number | Protein Names |
| --- | --- |
| A0A0G2JSK5 | Itgb1 - Integrin beta |
| A0A0G2JTL5 | Pc - Pyruvate carboxylase, mitochondrial |
| A0A0G2JTW9 | Hbb-b1 - Hemoglobin, beta adult major chain |
| A0A0G2JVH4 | Immt - MICOS complex subunit MIC60 |
| A0A0G2K401 | Pcca - Propionyl-CoA carboxylase alpha chain, mitochondrial |
| A0A0G2K531 | Gpx3 - Glutathione peroxidase |
| D3Z8D7 | Rps26 - ribosomal protein S26 |
| A0A0G2K8Q8 | Uqcr10 - Uncharacterized protein |
| A0A0G2KAM3 | Pdhb - Pyruvate dehydrogenase E1 component subunit beta, mitochondrial |
| A0A0G2KB63 | Phb2 - Prohibitin-2 |
| B2RZ24 | Sucla2 - succinyl-CoA ligase |
| F1LRJ9 | Selenbp1 - Selenium-binding protein 1 |
| G3V7I0 | Prdx3 - thioredoxin-dependent peroxide reductase |
| Q5BJ93 | Eno1 - Alpha-enolase |
| M0RAM5 | Gpx1 - Glutathione peroxidase |
| M0RBF1 | C3 - complement C3 precursor |
| P04639 | Apoa1 - Apolipoprotein A-I |
| P04797 | Gapdh - Glyceraldehyde-3-phosphate dehydrogenase |
| P10888 | Cox4i1 - Cytochrome c oxidase subunit 4 isoform 1 |
| P13803 | Etfa - Electron transfer flavoprotein subunit alpha |
| P14046 | A1i3 - alpha-1-inhibitor 3 precursor |
| P14408 | Fh - fumarate hydratase, mitochondrial precursor |
| Q6IMX3 | Acads - Short-chain specific acyl-CoA dehydrogenase |
| P17764 | Acat1 - Acetyl-CoA acetyltransferase |
| P18163 | Acsl1 - Long-chain-fatty-acid--CoA ligase 1 |
| P97601 | Hspe1 - 10 kDa heat shock protein |
| P32551 | Uqcrc2 - Cytochrome b-c1 complex subunit 2 |
| P54313 | Gnb2 - Guanine nucleotide-binding protein G(I)/G(S)/G(T) subunit beta-2 |
| P67779 | Phb - Prohibitin |
| P68255 | Ywhaq - 14-3-3 protein theta |
| P69897 | Tubb5 - Tubulin beta-5 chain |
| Q03336 | Rgn - Regucalcin |
| Q5BJZ3 | Nnt - NAD(P) transhydrogenase, mitochondrial |
| Q5M9H2 | Acadvl - very long-chain specific acyl-CoA dehydrogenase |
| Q5PQU1 | Kng1 - T-kininogen 2 precursor |
| Q5XI78 | Ogdh - 2-oxoglutarate dehydrogenase |
| Q641Y2 | Ndufs2 - NADH dehydrogenase [ubiquinone] iron-sulfur protein 2 |
| Q68FU3 | Etfb - Electron transfer flavoprotein subunit beta |
| Q6P6R2 | Dld - Dihydrolipoyl dehydrogenase |
| Q6P7P5 | Bzw1 - Basic leucine zipper and W2 domain-containing protein 1 |
| Q6P9V6 | Psma5 - proteasome subunit alpha type-5 |
| Q920L2 | Sdha - Succinate dehydrogenase [ubiquinone] flavoprotein subunit |
| Q9ER34 | Aco2 - Aconitate hydratase |
| Q9JJ54 | Hnrpd - Heterogeneous nuclear ribonucleoprotein C |

**Table D: *Comparative list of proteins in the aorta of insulin-treated diabetic rats compared to diabetic rats.***

|  | Accession Number | Names | Abbreviation | Fold Change | p value |
| --- | --- | --- | --- | --- | --- |
| 1 | A0A0F7RQJ6 | D-Dopachrome Tautomerase | DDT | 11.92 | 0.011 |
| 2 | A0A0G2JSI0 | Flavin Containing Monooxygenase 3 | FMO3 | 0.07 | 0.002 |
| 3 | Z4YNW7 | Protein Phosphatase 2 Regulatory Subunit Bbeta | PPP2R2B | 4.49 | 0.016 |
| 4 | A0A0G2JSR0 | Voltage Dependent Anion Channel 3 | VDAC3 | 0.04 | 0.000 |
| 5 | A0A0G2JSS9 | Atlastin-3 | Atl3 | 0.16 | 0.022 |
| 6 | A0A0G2JSU4 | N-Myc Downstream Regulated Gene 2, Isoform CRA_B | Ndrg2 | 0.60 | 0.022 |
| 7 | A0A0G2JT00 | Cuta Divalent Cation Tolerance Homolog | CUTA | 2.58 | 0.046 |
| 8 | G3V9Q3 | Heterogeneous Nuclear Ribonucleoprotein H1 | Hnrnph1 | 0.20 | 0.009 |
| 9 | D3ZB30 | Polypyrimidine Tract Binding Protein 1 | PTBP1 | 0.17 | 0.038 |
| 10 | A0A0G2JUC7 | Dynactin Subunit 2 | DCTN2 | 0.18 | 0.040 |
| 11 | A0A0G2JV31 | X-Prolyl Aminopeptidase 1 | XPNPEP1 | 0.43 | 0.006 |
| 12 | Q6P7S0 | Pyruvate Kinase, Muscle | PKM | 0.68 | 0.042 |
| 13 | A0A0G2JVH4 | Inner Membrane Mitochondrial Protein | IMMT | 0.05 | 0.001 |
| 14 | D3ZKE6 | Sarcolemmal Membrane-Associated Protein | Slmap | 0.07 | 0.010 |
| 15 | A0A0G2JWC7 | Fermitin Family Member 2 | FERMT2 | 0.13 | 0.010 |
| 16 | A0A0G2JXC3 | Ribosomal Protein S21 | RPS21 | 11.16 | 0.033 |
| 17 | G3V9W6 | Aldehyde Dehydrogenase 3 Family Member A2 | ALDH3A2 | 5.27 | 0.010 |
| 18 | A0A0G2JYD0 | Dynein Heavy Chain 7, Axonemal | Dnah7 | 0.03 | 0.007 |
| 19 | Q6IMY8 | Heterogeneous Nuclear Ribonucleoprotein U | HNRNPU | 0.38 | 0.032 |
| 20 | A0A0G2JZM2 | Sec23 Homolog A, CoAt Complex II Component | Sec23a | 0.27 | 0.001 |
| 21 | F1LMC7 | Septin 7 | SEPT7 | 0.20 | 0.040 |
| 22 | A0A0G2K013 | Actinin Alpha 4 | ACTN4 | 0.10 | 0.001 |
| 23 | F1LUV9 | Neural Cell Adhesion Molecule 1 | NCAM1 | 3.68 | 0.005 |
| 24 | A0A0G2K0Q7 | Myosin Light Chain Kinase | MYLK | 0.09 | 0.017 |
| 25 | A0A0G2K1C0 | ARP3 Actin Related Protein 3 Homolog | ACTR3 | 0.19 | 0.015 |
| 26 | F1LQQ1 | Malic Enzyme 1 | ME1 | 13.38 | 0.003 |
| 27 | A0A0G2K506 | Lactadherin | Mfge8 | 3.20 | 0.040 |
| 28 | A0A0G2K531 | Glutathione Peroxidase 3 | GPX3 | 0.87 | 0.035 |
| 29 | A0A0G2K757 | Ribophorin II | RPN2 | 0.27 | 0.025 |
| 30 | A0A0G2K7M2 | RAD23 Homolog A, Nucleotide Excision Repair Protein | Rad23a | 8.13 | 0.014 |
| 31 | R9PXU6 | Vinculin | VCL | 0.31 | 0.007 |
| 32 | Q5U1Y3 | Filamin Binding LIM Protein 1 | FBLIM1 | 0.13 | 0.005 |
| 33 | Q5PQM7 | LIM Zinc Finger Domain Containing 2 | LIMS2 | 0.16 | 0.018 |
| 34 | A0A0G2KB63 | Prohibitin 2 | PHB2 | 0.12 | 0.036 |
| 35 | A0A0H2UHM3 | Haptoglobin | HP | 1.48 | 0.044 |
| 36 | A0A0H2UHM5 | Protein Disulfide Isomerase Family A Member 3 | PDIA3 | 0.64 | 0.023 |
| 37 | A0A0H2UHQ8 | 40S Ribosomal Protein S17 | Rps17 | 0.45 | 0.046 |
| 38 | A0A1B0GWT7 | Late Endosomal/Lysosomal Adaptor, MAPK And MTOR Activator 2 | Lamtor2 | 2.88 | 0.002 |
| 39 | A0JPJ7 | Obg-Like Atpase 1 | Ola1 | 1.45 | 0.046 |
| 40 | A1L1M0 | Protein Kinase Camp-Activated Catalytic Subunit Alpha | PRKACA | 0.53 | 0.044 |
| 41 | A2VD12 | Pre-B-Cell Leukemia Transcription Factor-Interacting Protein 1 | Pbxip1 | 0.81 | 0.046 |
| 42 | D4ACV3 | Histone H2A | Hist2h2ac | 0.20 | 0.020 |
| 43 | F1LXV3 | Serine/Threonine Kinase 26 | STK26 | 5.35 | 0.000 |
| 44 | B2GUZ5 | F-Actin-Capping Protein Subunit Alpha-1 | Capza1 | 0.24 | 0.010 |
| 45 | B2GV06 | 3-Oxoacid CoA-Transferase 1 | OXCT1 | 0.16 | 0.029 |
| 46 | B2RYK3 | Sepiapterin Reductase (7,8-Dihydrobiopterin:NADP+ Oxidoreductase) | SPR | 9.85 | 0.020 |
| 47 | B3DM95 | Parathymosin | Ptms | 19.12 | 0.043 |
| 48 | B5DF65 | Biliverdin Reductase B | BLVRB | 2.43 | 0.039 |
| 49 | C0KUC5 | LIM Zinc Finger Domain Containing 1 | LIMS1 | 0.30 | 0.039 |
| 50 | D3Z8I7 | Glutathione S-Transferase, Theta 3 | Gstt3 | 5.17 | 0.037 |
| 51 | D3ZCA0 | Proline Synthase Co-Transcribed Bacterial Homolog Protein | Prosc | 0.27 | 0.038 |
| 52 | D3ZF13 | NADH:Ubiquinone Oxidoreductase Subunit AB1 | NDUFAB1 | 5.94 | 0.018 |
| 53 | D3ZIC4 | Protein Phosphatase 1, Regulatory (Inhibitor) Subunit 12B | Ppp1r12b | 0.17 | 0.018 |
| 54 | D3ZQ25 | Fibulin 1 | FBLN1 | 0.21 | 0.003 |
| 55 | D3ZS58 | NADH:Ubiquinone Oxidoreductase Subunit A2 | NDUFA2 | 6.23 | 0.047 |
| 56 | D4A5L9 | Protein LOC679794 | LOC690675 | 3.82 | 0.006 |
| 57 | D4ADS6 | Integrator Complex Subunit 7 | Ints7 | 2.70 | 0.045 |
| 58 | F1LN42 | Tensin 1 | TNS1 | 0.08 | 0.008 |
| 59 | F1LN88 | Aldehyde Dehydrogenase 2 Family (Mitochondrial) | ALDH2 | 0.04 | 0.000 |
| 60 | F1LRT9 | Dynein Cytoplasmic 1 Heavy Chain 1 | DYNC1H1 | 0.10 | 0.042 |
| 61 | F1LRV4 | Heat Shock Protein Family A (Hsp70) Member 4 | HSPA4 | 0.07 | 0.007 |
| 62 | F1LTF8 | Laminin Subunit Alpha 4 | LAMA4 | 0.22 | 0.023 |
| 63 | F1M779 | Clathrin Heavy Chain | CLTC | 0.06 | 0.032 |
| 64 | F1M866 | Sorbin And SH3 Domain Containing 1 | SORBS1 | 0.30 | 0.003 |
| 65 | F1M978 | Inositol Monophosphatase 1 | IMPA1 | 0.08 | 0.001 |
| 66 | Q6P7A4 | Prosaposin | PSAP | 1.69 | 0.015 |
| 67 | Q8CHN5 | Epididymal Secretory Protein E1 Precursor | Npc2 | 11.08 | 0.000 |
| 68 | G3V624 | Coronin-1C | Coro1c | 0.06 | 0.003 |
| 69 | Q5EBC3 | Methylenetetrahydrofolate Dehydrogenase, Cyclohydrolase And Formyltetrahydrofolate Synthetase 1 | MTHFD1 | 0.21 | 0.024 |
| 70 | G3V6Y6 | Glycogen Phosphorylase B | PYGB | 0.13 | 0.001 |
| 71 | G3V7C6 | Tubulin Beta 4B Class Ivb | TUBB4B | 0.50 | 0.014 |
| 72 | G3V7F3 | WNT1 Inducible Signaling Pathway Protein 2 | WISP2 | 17.46 | 0.043 |
| 73 | G3V7Q7 | IQ Motif Containing Gtpase Activating Protein 1 | IQGAP1 | 0.05 | 0.012 |
| 74 | G3V7U4 | Lamin B1 | LMNB1 | 0.29 | 0.005 |
| 75 | G3V818 | Parvin Alpha | PARVA | 0.11 | 0.031 |
| 76 | G3V852 | Talin 1 | TLN1 | 0.15 | 0.024 |
| 77 | G3V8A5 | Maternal Embryonic Message 3 | Vps35 | 0.11 | 0.047 |
| 78 | G3V8C3 | Vimentin | VIM | 0.25 | 0.019 |
| 79 | G3V8L3 | Lamin A/C | LMNA | 0.04 | 0.038 |
| 80 | G3V940 | Coronin 1B | CORO1B | 0.23 | 0.033 |
| 81 | G3V9E3 | Caldesmon 1 | Cald1 | 0.04 | 0.031 |
| 82 | M0R557 | Cardiomyopathy Associated 5 | Cmya5 | 15.54 | 0.005 |
| 83 | O35814 | Stress Induced Phosphoprotein 1 | STIP1 | 0.39 | 0.019 |
| 84 | Q6IN22 | Cathepsin B | CTSB | 5.28 | 0.021 |
| 85 | P01015 | Angiotensinogen | AGT | 5.89 | 0.025 |
| 86 | P02651 | Apolipoprotein A4 | APOA4 | 0.07 | 0.039 |
| 87 | P02767 | Transthyretin | TTR | 3.26 | 0.032 |
| 88 | P04182 | Ornithine Aminotransferase | OAT | 0.12 | 0.029 |
| 89 | P04633 | Uncoupling Protein 1 | UCP1 | 17.28 | 0.036 |
| 90 | P04638 | Apolipoprotein A2 | APOA2 | 2.34 | 0.036 |
| 91 | P04785 | Prolyl 4-Hydroxylase Subunit Beta | P4HB | 0.59 | 0.030 |
| 92 | P05197 | Eukaryotic Translation Elongation Factor 2 | EEF2 | 0.27 | 0.036 |
| 93 | P05545 | Serine (Or Cysteine) Proteinase Inhibitor, Clade A, Member 3C | Serpina3c /Serpina3m | 1.67 | 0.015 |
| 94 | P05708 | Hexokinase 1 | HK1 | 0.40 | 0.036 |
| 95 | Q5RK05 | Matrix Gla Protein | MGP | 5.25 | 0.011 |
| 96 | Q6LCA5 | Protein Kinase Camp-Dependent Type I Regulatory Subunit Alpha | PRKAR1A | 0.51 | 0.047 |
| 97 | P0DN35 | NADH Dehydrogenase [Ubiquinone] 1 Beta Subcomplex Subunit 1 | Ndufb1 | 3.98 | 0.000 |
| 98 | P11507 | Atpase Sarcoplasmic/Endoplasmic Reticulum Ca2+ Transporting 2 | ATP2A2 | 0.07 | 0.000 |
| 99 | P11517 | Hemoglobin Subunit Beta- | LOC689064 | 5.18 | 0.035 |
| 100 | P12007 | Isovaleryl-CoA Dehydrogenase | IVD | 4.75 | 0.030 |
| 101 | P14046 | Murinoglobulin 1 | Mug1 (includes others) | 1.96 | 0.002 |
| 102 | P14668 | Annexin A5 | ANXA5 | 1.41 | 0.035 |
| 103 | P16636 | Lysyl Oxidase | LOX | 9.07 | 0.034 |
| 104 | Q6P9V5 | Proteasome Subunit Alpha 2 | PSMA2 | 0.68 | 0.017 |
| 105 | P20070 | Cytochrome B5 Reductase 3 | Cyb5r3 | 0.21 | 0.028 |
| 106 | Q4QRB8 | Argininosuccinate Lyase | ASL | 0.13 | 0.012 |
| 107 | P24329 | Thiosulfate Sulfurtransferase | TST | 0.20 | 0.032 |
| 108 | P25093 | Fumarylacetoacetate Hydrolase | FAH | 0.18 | 0.007 |
| 109 | P27867 | Sorbitol Dehydrogenase | SORD | 0.44 | 0.044 |
| 110 | P28480 | T-Complex 1 | TCP1 | 0.08 | 0.011 |
| 111 | P29266 | 3-Hydroxyisobutyrate Dehydrogenase | HIBADH | 0.19 | 0.002 |
| 112 | Q6P2A5 | Adenylate Kinase 3 | AK3 | 0.44 | 0.049 |
| 113 | P31430 | Dipeptidase 1 (Renal) | DPEP1 | 9.23 | 0.031 |
| 114 | P32198 | Carnitine Palmitoyltransferase 1A | CPT1A | 0.18 | 0.047 |
| 115 | P39069 | Adenylate Kinase 1 | AK1 | 1.55 | 0.001 |
| 116 | P46462 | Valosin Containing Protein | VCP | 0.16 | 0.007 |
| 117 | P47820 | Angiotensin I Converting Enzyme | ACE | 2.02 | 0.007 |
| 118 | P48508 | Glutamate-Cysteine Ligase Modifier Subunit | GCLM | 7.90 | 0.044 |
| 119 | P50398 | GDP Dissociation Inhibitor 1 | GDI1 | 0.66 | 0.048 |
| 120 | P54313 | G Protein Subunit Beta 2 | GNB2 | 0.52 | 0.019 |
| 121 | P62260 | Tyrosine 3-Monooxygenase/Tryptophan 5-Monooxygenase Activation Protein Epsilon | YWHAE | 0.44 | 0.028 |
| 122 | P69897 | Tubulin Beta Class I | TUBB | 0.49 | 0.001 |
| 123 | P82995 | Heat Shock Protein 90 Alpha Family Class A Member 1 | HSP90AA1 | 0.06 | 0.013 |
| 124 | P85515 | Alpha-Centractin | Actr1a | 0.28 | 0.000 |
| 125 | P85973 | Purine Nucleoside Phosphorylase | PNP | 0.42 | 0.012 |
| 126 | Q06647 | ATP Synthase, H+ Transporting, Mitochondrial F1 Complex, O Subunit | ATP5O | 0.21 | 0.019 |
| 127 | Q08163 | Adenylate Cyclase Associated Protein 1 | CAP1 | 0.15 | 0.024 |
| 128 | Q4V8H5 | Aspartyl Aminopeptidase | Dnpep | 0.09 | 0.011 |
| 129 | Q4V8H8 | EH Domain-Containing Protein 2 | Ehd2 | 0.15 | 0.003 |
| 130 | Q5BJZ3 | Nicotinamide Nucleotide Transhydrogenase | NNT | 0.13 | 0.004 |
| 131 | Q5FVG5 | Tropomyosin 2, Beta | Tpm2 | 0.06 | 0.028 |
| 132 | Q5I0P2 | Glycine Cleavage System Protein H | GCSH | 4.28 | 0.042 |
| 133 | Q5RJR8 | Leucine-Rich Repeat-Containing Protein 59 | Lrrc59 | 0.45 | 0.026 |
| 134 | Q5UAJ6 | Cytochrome C Oxidase Subunit II | MT-CO2 | 4.38 | 0.003 |
| 135 | Q5XI21 | Target of Myb Protein 1 | Tom1 | 5.51 | 0.001 |
| 136 | Q5XI34 | Protein Phosphatase 2 Scaffold Subunit Aalpha | PPP2R1A | 0.56 | 0.021 |
| 137 | Q5XIH1 | Asporin Precursor | Aspn | 0.48 | 0.012 |
| 138 | Q63413 | Dexd-Box Helicase 39B | DDX39B | 0.09 | 0.014 |
| 139 | Q63570 | Proteasome 26S Subunit, Atpase 4 | PSMC4 | 1.54 | 0.030 |
| 140 | Q63610 | Tropomyosin 3 | Tpm3 | 0.05 | 0.001 |
| 141 | Q64240 | Alpha-1-Microglobulin/Bikunin Precursor | AMBP | 0.44 | 0.019 |
| 142 | Q68FR9 | Eukaryotic Translation Elongation Factor 1 Delta | EEF1D | 0.09 | 0.006 |
| 143 | Q68FT3 | Pyridine Nucleotide-Disulfide Oxidoreductase Domain-Containing Protein 2 | Pyroxd2 | 2.53 | 0.034 |
| 144 | Q6AYQ4 | Transmembrane Protein 109 | Tmem109 | 0.15 | 0.031 |
| 145 | Q6AYT0 | Quinone Oxidoreductase | Cryz | 1.73 | 0.012 |
| 146 | Q6GMN8 | Actinin Alpha 1 | ACTN1 | 0.08 | 0.000 |
| 147 | Q6P502 | Chaperonin Containing TCP1 Subunit 3 | CCT3 | 0.19 | 0.009 |
| 148 | Q6P725 | Desmin | DES | 0.09 | 0.042 |
| 149 | Q6P7A7 | Dolichyl-Diphosphooligosaccharide--Protein Glycosyltransferase Subunit 1 Precursor | Rpn1 | 0.09 | 0.022 |
| 150 | Q6P7P5 | Basic Leucine Zipper and W2 Domain-Containing Protein 1 | Bzw1 | 6.22 | 0.002 |
| 151 | Q6P9V9 | Tubulin Alpha-1A Chain | Tuba1b | 0.46 | 0.021 |
| 152 | Q794E4 | Heterogeneous Nuclear Ribonucleoprotein F | HNRNPF | 0.55 | 0.034 |
| 153 | Q811X6 | Crystallin Lambda 1 | CRYL1 | 0.32 | 0.017 |
| 154 | Q91Y81 | Septin 2 | SEPT2 | 0.16 | 0.026 |
| 155 | Q920A6 | Serine Carboxypeptidase 1 | SCPEP1 | 2.14 | 0.006 |
| 156 | Q923Z2 | Tropomyosin 1, Alpha | Tpm1 | 0.23 | 0.021 |
| 157 | Q99PD6 | Transforming Growth Factor Beta 1 Induced Transcript 1 | TGFB1I1 | 0.09 | 0.011 |
| 158 | Q9ESN0 | Family with Sequence Similarity 129 Member A | FAM129A | 0.07 | 0.045 |
| 159 | Q9JJ54 | Heterogeneous Nuclear Ribonucleoprotein D | HNRNPD | 0.19 | 0.009 |
| 160 | Q9QZQ5 | Nephroblastoma Overexpressed | NOV | 3.40 | 0.033 |
| 161 | Q9Z1H9 | Protein Kinase C Delta-Binding Protein | Prkcdbp | 0.09 | 0.043 |
| 162 | Q9Z1X1 | Extended Synaptotagmin-1 | Esyt1 | 0.08 | 0.003 |
| 163 | Q9Z2G3 | ATP Citrate Lyase | ACLY | 10.51 | 0.017 |

**Table E: *Comparative list of proteins in the kidney of insulin-treated diabetic rats compared to diabetic rats.***

|  | Accession Number | Names | Abbreviation | Fold Change | p value |
| --- | --- | --- | --- | --- | --- |
| 1 | A0A096MK30 | Moesin | MSN | 1.21 | 0.004 |
| 2 | Q8SEZ2 | Cytochrome C Oxidase Subunit 3 | Mt-cox3 | 0.94 | 0.037 |
| 3 | A0A0A0MXW3 | H2A Histone Family Member Z | H2AFZ | 1.30 | 0.049 |
| 4 | Q95938 | Cytochrome C Oxidase Subunit 1 | Mt-co1 | 0.85 | 0.026 |
| 5 | Q9Z1J7 | Solute Carrier Family 1 Member 5 | SLC1A5 | 2.45 | 0.006 |
| 6 | A0A0G2JSK5 | Integrin Subunit Beta 1 | ITGB1 | 1.26 | 0.047 |
| 7 | A0A0G2JSS8 | Peroxiredoxin 5 | PRDX5 | 0.81 | 0.023 |
| 8 | A0A0G2JSV6 | Hemoglobin, Alpha 1 | Hba1/Hba2 | 2.48 | 0.039 |
| 9 | A0A0G2JTH4 | CD47 Molecule | CD47 | 1.30 | 0.020 |
| 10 | A0A0G2JTL5 | Pyruvate Carboxylase | PC | 0.57 | 0.005 |
| 11 | Q66WT9 | Phosphatidylinositol Binding Clathrin Assembly Protein | PICALM | 2.11 | 0.001 |
| 12 | A0A0G2JTW9 | Hemoglobin Subunit Beta | HBB | 3.00 | 0.023 |
| 13 | D4A6E3 | Murinoglobulin-1 Precursor | Mug1 | 3.05 | 0.018 |
| 14 | F1LPD0 | Collagen Type XV Alpha 1 Chain | Col15a1 | 9.11 | 0.017 |
| 15 | D4A1H0 | ATPase H+ Transporting V0 Subunit A4 | ATP6V0A4 | 1.23 | 0.008 |
| 16 | A0A0G2JWC7 | Fermitin Family Member 2 | FERMT2 | 1.20 | 0.039 |
| 17 | M0R4L7 | Histone H2B | Hist1h2bl | 1.39 | 0.020 |
| 18 | D4A554 | Eukaryotic Translation Initiation Factor 4 Gamma 3 | EIF4G3 | 4.73 | 0.039 |
| 19 | A0A0G2K4D0 | Ankyrin 3 | ANK3 | 1.14 | 0.036 |
| 20 | A0A0G2K0Q8 | Sushi Domain-Containing 2 | Susd2 | 0.47 | 0.001 |
| 21 | A0A0G2K277 | Enoyl-Coenzyme A Delta Isomerase 3 | Eci3 | 0.72 | 0.000 |
| 22 | B0BMT6 | RCG43995, Isoform CRA_B | Tpmt | 1.22 | 0.032 |
| 23 | A0A0G2K654 | Histone Cluster 1 H1 Family Member C | Hist1h1c | 1.50 | 0.005 |
| 24 | A0A0G2K6H2 | Glutathione S-Transferase Zeta 1 | GSTZ1 | 0.70 | 0.014 |
| 25 | D3ZCR3 | High Mobility Group Box 1 | Gm21596 /Hmgb1 | 1.57 | 0.049 |
| 26 | A0A0G2K737 | Thioredoxin Like 1 | TXNL1 | 1.24 | 0.012 |
| 27 | D3Z8D7 | 40S Ribosomal Protein S26 | LOC100361854 | 1.23 | 0.030 |
| 28 | F1LS02 | Nucleoporin 155 | NUP155 | 2.75 | 0.045 |
| 29 | A0A0G2K8Q8 | Ubiquinol-Cytochrome C Reductase, Complex III Subunit X | UQCR10 | 0.88 | 0.024 |
| 30 | Q80YG1 | Phosphoserine Aminotransferase 1 | PSAT1 | 1.52 | 0.013 |
| 31 | G3V7K3 | Ceruloplasmin | CP | 4.30 | 0.019 |
| 32 | A0A0G2K9W7 | LDL Receptor Related Protein 2 | LRP2 | 2.84 | 0.006 |
| 33 | Q7TNX2 | Liver Regeneration-Related Protein LRRG01 | Pxmp2 | 0.56 | 0.010 |
| 34 | A0A0H2UHE4 | Regenerating Family Member 3 Alpha | REG3A | 0.13 | 0.023 |
| 35 | A0A0H2UHM3 | Haptoglobin | HP | 1.77 | 0.010 |
| 36 | B1WBN9 | Pyruvate Kinase, Liver And RBC | PKLR | 1.65 | 0.029 |
| 37 | A0JPM9 | Eukaryotic Translation Initiation Factor 3 Subunit J | EIF3J | 0.30 | 0.011 |
| 38 | A2VCW9 | Aminoadipate-Semialdehyde Synthase | AASS | 0.36 | 0.001 |
| 39 | A9UMW1 | Glutathione S-Transferase, Alpha 4 | Gsta4 | 0.60 | 0.019 |
| 40 | B0BMW2 | Hydroxysteroid 17-Beta Dehydrogenase 10 | HSD17B10 | 0.70 | 0.020 |
| 41 | B0BNG1 | Proline Dehydrogenase 2 | PRODH2 | 0.78 | 0.028 |
| 42 | B0BNN3 | Carbonic Anhydrase 1 | CA1 | 4.95 | 0.047 |
| 43 | B1WC26 | N-Acetylneuraminate Synthase | NANS | 1.15 | 0.027 |
| 44 | B2GVB1 | S100 Calcium Binding Protein A6 | S100A6 | 0.76 | 0.029 |
| 45 | B2RYR8 | Ribosomal Protein S8 | RPS8 | 1.33 | 0.017 |
| 46 | B2RYW9 | Fumarylacetoacetate Hydrolase Domain-Containing Protein 2 | Fahd2 | 0.55 | 0.008 |
| 47 | B4F768 | Aldehyde Dehydrogenase 4 Family Member A1 | ALDH4A1 | 0.54 | 0.018 |
| 48 | B5DF36 | Placenta-Specific 8 | Plac8 | 1.56 | 0.019 |
| 49 | B5DFA0 | Vil1 Protein | Vil1 | 1.41 | 0.009 |
| 50 | B6DYQ0 | Glutathione S-Transferase Kappa 1 | GSTK1 | 0.58 | 0.047 |
| 51 | B6DYQ7 | Glutathione S-Transferase Pi 1 | GSTP1 | 1.38 | 0.030 |
| 52 | D3Z8F1 | Villin-Like | Vill | 1.42 | 0.048 |
| 53 | D3ZD09 | Cytochrome C Oxidase Subunit 6B1 | COX6B1 | 0.79 | 0.046 |
| 54 | F1LZC5 | Protein Ndufa13 | Ndufa13 | 0.77 | 0.008 |
| 55 | O35802 | Inter-Alpha-Trypsin Inhibitor Heavy Chain Family Member 4 | ITIH4 | 5.05 | 0.026 |
| 56 | D3ZFJ6 | Lactamase, Beta | Lactb | 0.79 | 0.043 |
| 57 | D3ZFY8 | Ubiquitin-Conjugating Enzyme E2 Variant 1-Like | LOC100912618 | 1.27 | 0.043 |
| 58 | D3ZG43 | NADH:Ubiquinone Oxidoreductase Core Subunit S3 | NDUFS3 | 0.77 | 0.017 |
| 59 | D3ZT90 | Glutaryl-CoA Dehydrogenase | GCDH | 0.63 | 0.003 |
| 60 | D3ZTX4 | Aldehyde Dehydrogenase 8 Family Member A1 | ALDH8A1 | 3.17 | 0.000 |
| 61 | D3ZXY4 | Aldehyde Dehydrogenase 8 Family, Member A1 | Aldh8a1 | 3.07 | 0.000 |
| 62 | D3ZZN3 | Acyl-CoA Synthetase Short-Chain Family Member 1 | ACSS1 | 0.62 | 0.005 |
| 63 | D4A269 | Uncharacterized Protein | N/A | 1.13 | 0.017 |
| 64 | D4A5I9 | Myosin VI | MYO6 | 1.18 | 0.018 |
| 65 | D4A5L9 | Protein LOC679794 | LOC679794 | 1.67 | 0.004 |
| 66 | F1LTW8 | Similar To Ribosomal Protein S23 | RGD1563705 | 1.21 | 0.042 |
| 67 | F1LMF4 | Protocadherin Fat 3 | Fat3 | 2.46 | 0.031 |
| 68 | Q63654 | Ubiquitin B | Ubb | 1.10 | 0.018 |
| 69 | F1LMP9 | DAB2, Clathrin Adaptor Protein | DAB2 | 1.52 | 0.014 |
| 70 | F1LMV6 | Desmoplakin | DSP | 1.31 | 0.024 |
| 71 | F1LN88 | Aldehyde Dehydrogenase 2 Family (Mitochondrial) | ALDH2 | 0.82 | 0.030 |
| 72 | F1LP30 | Methylcrotonoyl-CoA Carboxylase 1 | MCCC1 | 0.72 | 0.027 |
| 73 | F1LPD6 | Acetyl-CoA Acyltransferase 1 | ACAA1 | 0.64 | 0.006 |
| 74 | F1LRJ9 | Selenium-Binding Protein 1 | Selenbp1 | 0.64 | 0.016 |
| 75 | F1LRY5 | Sarcosine Dehydrogenase | SARDH | 0.63 | 0.028 |
| 76 | F1LWL8 | Solute Carrier Family 5 Member 12 | Slc5a12 | 0.79 | 0.025 |
| 77 | F1LZW6 | Solute Carrier Family 25 Member 13 | Slc25a13 | 1.17 | 0.038 |
| 78 | F1M949 | Cytoskeleton Associated Protein 5 | CKAP5 | 0.21 | 0.009 |
| 79 | F1MAA7 | Laminin Subunit Gamma 1 | LAMC1 | 2.53 | 0.035 |
| 80 | Q5BKC4 | Complement C9 | C9 | 8.19 | 0.002 |
| 81 | G3V6D3 | ATP Synthase, H+ Transporting, Mitochondrial F1 Complex, Beta Polypeptide | ATP5B | 0.86 | 0.008 |
| 82 | G3V741 | Solute Carrier Family 25 Member 3 | SLC25A3 | 0.89 | 0.044 |
| 83 | G3V7I0 | Peroxiredoxin 3 | PRDX3 | 0.76 | 0.028 |
| 84 | G3V7J0 | Aldehyde Dehydrogenase 6 Family Member A1 | ALDH6A1 | 0.88 | 0.043 |
| 85 | G3V7Q7 | IQ Motif Containing Gtpase Activating Protein 1 | IQGAP1 | 1.27 | 0.007 |
| 86 | G3V852 | Talin 1 | TLN1 | 1.40 | 0.017 |
| 87 | G3V8C4 | Chloride Intracellular Channel 4 | CLIC4 | 1.30 | 0.034 |
| 88 | G3V960 | Guanidinoacetate N-Methyltransferase | GAMT | 1.89 | 0.017 |
| 89 | G3V9V9 | Cysteine And Glycine Rich Protein 2 | CSRP2 | 1.88 | 0.015 |
| 90 | M0R3V4 | Myeloid-Derived Growth Factor | Mydgf | 3.94 | 0.038 |
| 91 | Q5BJ93 | Enolase 1 | ENO1 | 1.21 | 0.003 |
| 92 | M0RAM5 | Glutathione Peroxidase 1 | GPX1 | 0.68 | 0.012 |
| 93 | M0RBF1 | Complement C3 | C3 | 2.11 | 0.026 |
| 94 | M0RDH0 | Glycine N-Methyltransferase | Gnmt | 0.77 | 0.008 |
| 95 | O35078 | D-Amino Acid Oxidase | DAO | 0.78 | 0.015 |
| 96 | O35509 | RAB11B, Member RAS Oncogene Family | RAB11B | 1.17 | 0.005 |
| 97 | P00173 | Cytochrome B5 Type A | CYB5A | 0.75 | 0.017 |
| 98 | P00507 | Glutamic-Oxaloacetic Transaminase 2 | GOT2 | 0.69 | 0.007 |
| 99 | P02761 | Alpha-2u-Globulin (L Type) Precursor | Mup5 | 2.63 | 0.002 |
| 100 | P02770 | Albumin | ALB | 1.63 | 0.001 |
| 101 | Q45QN0 | G Protein Subunit Alpha I2 | GNAI2 | 1.61 | 0.012 |
| 102 | P05065 | Aldolase, Fructose-Bisphosphate A | ALDOA | 1.18 | 0.028 |
| 103 | P05545 | Serine (Or Cysteine) Proteinase Inhibitor, Clade A, Member 3C | Serpina3c /Serpina3m | 2.39 | 0.018 |
| 104 | P07171 | Calbindin 1 | CALB1 | 0.48 | 0.010 |
| 105 | P07314 | Gamma-Glutamyltransferase 1 | GGT1 | 1.62 | 0.018 |
| 106 | P07687 | Epoxide Hydrolase 1 | EPHX1 | 0.39 | 0.004 |
| 107 | P07895 | Superoxide Dismutase 2 | SOD2 | 0.82 | 0.001 |
| 108 | P09034 | Argininosuccinate Synthase 1 | ASS1 | 0.77 | 0.024 |
| 109 | P10860 | Glutamate Dehydrogenase 1 | GLUD1 | 0.78 | 0.005 |
| 110 | P10888 | Cytochrome C Oxidase Subunit 4I1 | COX4I1 | 0.75 | 0.032 |
| 111 | P12346 | Transferrin | TF | 1.61 | 0.003 |
| 112 | P13084 | Nucleophosmin | NPM1 | 1.17 | 0.033 |
| 113 | P13803 | Electron Transfer Flavoprotein Alpha Subunit | ETFA | 0.81 | 0.019 |
| 114 | P14046 | Murinoglobulin 1 | Mug1 (includes others) | 4.72 | 0.000 |
| 115 | P14173 | DOPA Decarboxylase | DDC | 0.66 | 0.036 |
| 116 | P14480 | Fibrinogen Beta Chain | FGB | 2.50 | 0.039 |
| 117 | P14604 | Enoyl-CoA Hydratase, Short Chain 1 | ECHS1 | 0.79 | 0.040 |
| 118 | P16617 | Phosphoglycerate Kinase 1 | PGK1 | 0.88 | 0.030 |
| 119 | P17764 | Acetyl-CoA Acetyltransferase 1 | ACAT1 | 0.84 | 0.043 |
| 120 | Q66HM2 | Adaptor Related Protein Complex 2 Alpha 2 Subunit | AP2A2 | 1.19 | 0.036 |
| 121 | P19112 | Fructose-Bisphosphatase 1 | FBP1 | 1.96 | 0.001 |
| 122 | P20059 | Hemopexin | HPX | 1.25 | 0.034 |
| 123 | Q04970 | Neuroblastoma RAS Viral Oncogene Homolog | NRAS | 2.76 | 0.021 |
| 124 | P21913 | Succinate Dehydrogenase Complex Iron Sulfur Subunit B | SDHB | 0.80 | 0.026 |
| 125 | Q68G41 | Enoyl-CoA Delta Isomerase 1 | ECI1 | 0.62 | 0.008 |
| 126 | P27605 | Hypoxanthine Phosphoribosyltransferase 1 | HPRT1 | 0.65 | 0.015 |
| 127 | Q6P2A5 | Adenylate Kinase 3 | AK3 | 0.70 | 0.026 |
| 128 | P32755 | 4-Hydroxyphenylpyruvate Dioxygenase | HPD | 0.59 | 0.001 |
| 129 | P35213 | Tyrosine 3-Monooxygenase/Tryptophan 5-Monooxygenase Activation Protein Beta | YWHAB | 0.88 | 0.025 |
| 130 | P35467 | S100 Calcium Binding Protein A1 | S100A1 | 0.78 | 0.009 |
| 131 | P36511 | UDP Glucuronosyltransferase Family 2 Member B7 | UGT2B7 | 0.60 | 0.044 |
| 132 | P38918 | Aldo-Keto Reductase Family 7 Member A3 | AKR7A3 | 1.41 | 0.019 |
| 133 | P42123 | Lactate Dehydrogenase B | LDHB | 0.73 | 0.019 |
| 134 | P43428 | Glucose-6-Phosphatase Catalytic Subunit | G6PC | 0.73 | 0.033 |
| 135 | P50137 | Transketolase | TKT | 0.88 | 0.031 |
| 136 | P50554 | 4-Aminobutyrate Aminotransferase | ABAT | 0.73 | 0.036 |
| 137 | P56574 | Isocitrate Dehydrogenase (NADP(+)) 2, Mitochondrial | IDH2 | 0.90 | 0.031 |
| 138 | P61459 | Pterin-4 Alpha-Carbinolamine Dehydratase 1 | PCBD1 | 0.78 | 0.008 |
| 139 | P62076 | Mitochondrial Import Inner Membrane Translocase Subunit Tim13 | Timm13 | 0.81 | 0.048 |
| 140 | P62749 | Hippocalcin-Like Protein 1 | Hpcal1 | 1.29 | 0.039 |
| 141 | P62804 | Histone H4 Osteogenic Growth Peptide | Hist2h4 | 1.40 | 0.007 |
| 142 | P63018 | Heat Shock Protein Family A (Hsp70) Member 8 | HSPA8 | 1.10 | 0.023 |
| 143 | P63259 | Actin Gamma 1 | ACTG1 | 1.09 | 0.029 |
| 144 | P68035 | Actin, Alpha, Cardiac Muscle 1 | ACTC1 | 1.45 | 0.038 |
| 145 | P68182 | Protein Kinase Camp-Activated Catalytic Subunit Beta | PRKACB | 2.71 | 0.042 |
| 146 | P68511 | Tyrosine 3-Monooxygenase/Tryptophan 5-Monooxygenase Activation Protein Eta | YWHAH | 1.20 | 0.024 |
| 147 | P69897 | Tubulin Beta Class I | TUBB | 1.28 | 0.007 |
| 148 | P70473 | Alpha-Methylacyl-CoA Racemase | AMACR | 0.43 | 0.000 |
| 149 | Q66HP8 | Solute Carrier Family 25 Member 20 | SLC25A20 | 0.77 | 0.007 |
| 150 | Q03336 | Regucalcin | RGN | 2.16 | 0.016 |
| 151 | Q3B8N9 | Biphenyl Hydrolase-Like | Bphl | 0.77 | 0.008 |
| 152 | Q3T1K5 | Capping Actin Protein Of Muscle Z-Line Alpha Subunit 2 | CAPZA2 | 1.43 | 0.033 |
| 153 | Q4QQW3 | Alcohol Dehydrogenase, Iron Containing 1 | ADHFE1 | 0.29 | 0.002 |
| 154 | Q4V8F6 | Poly(Rc)-Binding Protein 2 | Pcbp2 | 1.75 | 0.042 |
| 155 | Q4V8H5 | Aspartyl Aminopeptidase | Dnpep | 0.69 | 0.018 |
| 156 | Q562C4 | Methyltransferase-Like Protein 7B | Mettl7b | 0.75 | 0.022 |
| 157 | Q5BJT9 | Creatine Kinase, Mitochondrial 1B | CKMT1A /CKMT1B | 0.67 | 0.018 |
| 158 | Q5BJZ3 | Nicotinamide Nucleotide Transhydrogenase | NNT | 0.66 | 0.025 |
| 159 | Q5DT04 | UDP Glucuronosyltransferase Family 1 Member A1 | UGT1A1 | 1.62 | 0.020 |
| 160 | Q5I0K3 | Citrate Lyase Subunit Beta-Like Protein | Clybl | 3.62 | 0.009 |
| 161 | Q5RJN0 | NADH:Ubiquinone Oxidoreductase Core Subunit S7 | NDUFS7 | 0.65 | 0.042 |
| 162 | Q5RKI0 | WD Repeat-Containing Protein 1 | Wdr1 | 1.26 | 0.025 |
| 163 | Q5U3Z7 | Serine Hydroxymethyltransferase 2 | SHMT2 | 0.78 | 0.007 |
| 164 | Q5UAJ5 | ATP Synthase F0 Subunit 8 (Mitochondrion) | ATP8 | 0.79 | 0.044 |
| 165 | Q5XI78 | Oxoglutarate Dehydrogenase | OGDH | 0.78 | 0.021 |
| 166 | Q5XI85 | Aminomethyltransferase | AMT | 0.60 | 0.005 |
| 167 | Q5XIF6 | Tubulin Alpha 4a | TUBA4A | 1.37 | 0.026 |
| 168 | Q63010 | Liver Carboxylesterase B-1 Precursor | Ces1f | 0.73 | 0.009 |
| 169 | Q63025 | Alpha-2u-Globulin (L Type) Precursor | Mup5 | 245.82 | 0.040 |
| 170 | Q63342 | Dimethylglycine Dehydrogenase | DMGDH | 0.67 | 0.001 |
| 171 | Q63413 | Spliceosome RNA Helicase Ddx39b | Ddx39b | 1.32 | 0.033 |
| 172 | Q63910 | Hemoglobin Subunit Alpha 2 | HBA1/HBA2 | 5.54 | 0.033 |
| 173 | Q64057 | Aldehyde Dehydrogenase 7 Family Member A1 | ALDH7A1 | 0.81 | 0.026 |
| 174 | Q641Y2 | NADH:Ubiquinone Oxidoreductase Core Subunit S2 | NDUFS2 | 0.75 | 0.016 |
| 175 | Q64319 | Solute Carrier Family 3 Member 1 | SLC3A1 | 1.72 | 0.050 |
| 176 | Q66H12 | Alpha-N-Acetylgalactosaminidase | Naga | 0.71 | 0.011 |
| 177 | Q66HF1 | NADH:Ubiquinone Oxidoreductase Core Subunit S1 | NDUFS1 | 0.79 | 0.015 |
| 178 | Q66HF3 | Electron Transfer Flavoprotein-Ubiquinone Oxidoreductase | Etfdh | 0.74 | 0.011 |
| 179 | Q68FR9 | Eukaryotic Translation Elongation Factor 1 Delta | EEF1D | 1.42 | 0.028 |
| 180 | Q68G31 | Phenazine Biosynthesis-Like Domain-Containing Protein | Pbld1 | 0.76 | 0.013 |
| 181 | Q6AYS2 | Sideroflexin 1 | SFXN1 | 0.71 | 0.017 |
| 182 | Q6I7R1 | Dehydrogenase/Reductase (SDR Family) Member 7 | Dhrs7l1 | 1.90 | 0.028 |
| 183 | Q6JE36 | Protein NDRG1 | Ndrg1 | 0.76 | 0.015 |
| 184 | Q9WVJ6 | Transglutaminase 2 | TGM2 | 1.63 | 0.026 |
| 185 | Q6P9V9 | Hydroxysteroid 11-Beta Dehydrogenase 1 | HSD11B1 | 1.21 | 0.034 |
| 186 | Q812B0 | Succinate Dehydrogenase [Ubiquinone] Cytochrome B Small Subunit | Lrrp1 | 0.78 | 0.031 |
| 187 | Q6TUH9 | Corticosteroid 11-Beta-Dehydrogenase Isozyme 1 | Hsd11b1 | 1.55 | 0.012 |
| 188 | Q6ZMA0 | Olfactory Receptor | Olr1868 | 0.58 | 0.021 |
| 189 | Q7TP52 | Carboxymethylenebutenolidase Homolog | Cmbl | 0.79 | 0.031 |
| 190 | Q811X6 | Crystallin Lambda 1 | CRYL1 | 0.70 | 0.011 |
| 191 | Q9JJI4 | Alpha-2u-Globulin (L Type) Precursor | Mup5 | 4.34 | 0.002 |
| 192 | Q8VI04 | Asparaginase Like 1 | ASRGL1 | 1.35 | 0.043 |
| 193 | Q91ZW6 | Trimethyllysine Hydroxylase, Epsilon | TMLHE | 0.35 | 0.029 |
| 194 | Q920A6 | Serine Carboxypeptidase 1 | SCPEP1 | 1.41 | 0.000 |
| 195 | Q920L2 | Succinate Dehydrogenase Complex Flavoprotein Subunit A | SDHA | 0.78 | 0.038 |
| 196 | Q923Z2 | Tropomyosin 1, Alpha | Tpm1 | 1.25 | 0.013 |
| 197 | Q9EPB1 | Dipeptidyl Peptidase 2 | Dpp7 | 1.37 | 0.027 |
| 198 | Q9EQS0 | Transaldolase 1 | TALDO1 | 1.40 | 0.006 |
| 199 | Q9ER34 | Aconitase 2 | ACO2 | 0.72 | 0.004 |
| 200 | Q9JJ54 | Heterogeneous Nuclear Ribonucleoprotein D | HNRNPD | 1.28 | 0.007 |
| 201 | Q9QX71 | Napsin A Aspartic Peptidase Precursor | Napsa | 0.86 | 0.043 |
| 202 | Q9QYU4 | Crystallin Mu | CRYM | 0.51 | 0.016 |
| 203 | Q9Z2L0 | Voltage Dependent Anion Channel 1 | VDAC1 | 0.87 | 0.015 |
| 204 | Q9Z2M4 | Peroxisomal 2,4-Dienoyl-CoA Reductase | Decr2 | 0.60 | 0.008 |

Supplementary Figure Legend:

**Figure A:** Histograms of intensity frequency in and aorta and kidney samples. Log (base 2)-transformed label-free quantification (LFQ) intensities of proteins in each sample were presented in individual histograms, showing the distribution of LFQ intensities. A: LFQ of the aorta control samples. B: LFQ of the aorta diabetes samples. C: LFQ of the aorta insulin-treated diabetes samples. D: LFQ of the kidney control samples. E: LFQ of the kidney diabetes samples. F: LFQ of the kidney insulin-treated diabetes samples.

**Figure B:** IHC staining of TGFβ levels in the aorta of the different rat groups. The intensity of TGFβ staining relative to Hoechst staining was assessed and represented in the bar graph. TGFβ staining was significantly increased in diabetic rats compared to non-diabetic control rats (2.5±0.39-fold, diabetes vs. control, * p=0.026, n=3). On the other hand, insulin supplementation significantly decreased TGFβ levels (0.9±0.046-fold, n=3, # p=0.014 Insulin vs. diabetes) relative to the diabetic group.

**Figure C:** IHC staining of cofilin1 levels in the kidney cortices of the different rat groups. The intensity of cofilin staining relative to Hoechst staining was assessed and represented in the bar graph. Coflin1 staining was significantly decreased in both diabetic and insulin-treated diabetic rats compared to non-diabetic control rats (0.4±0.05-fold, diabetes vs. control, *p=0.007, n=3; 0.5±0.06-fold, insulin-treated diabetic vs control, *p=0.012, n=3 respectively).


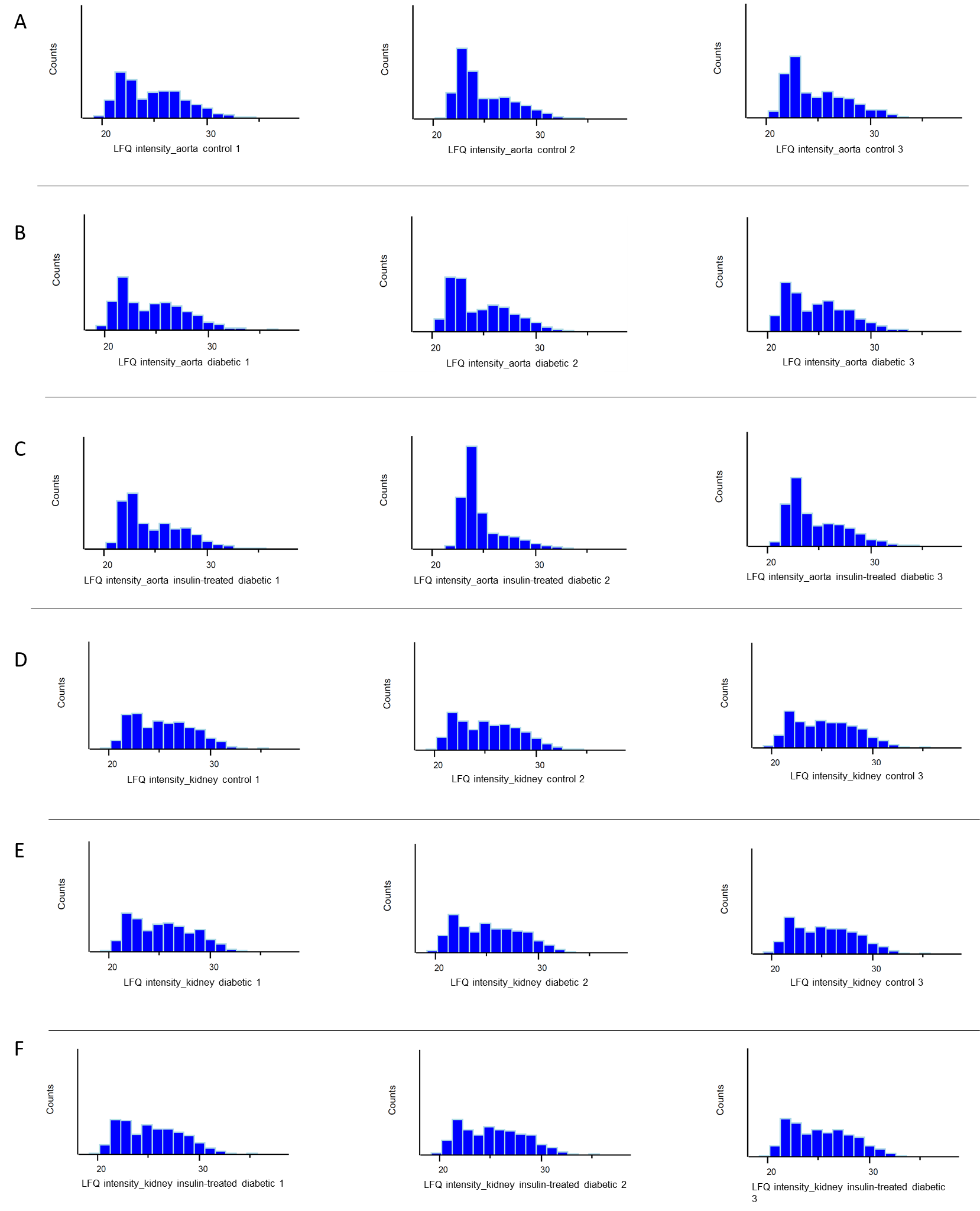


Figure A


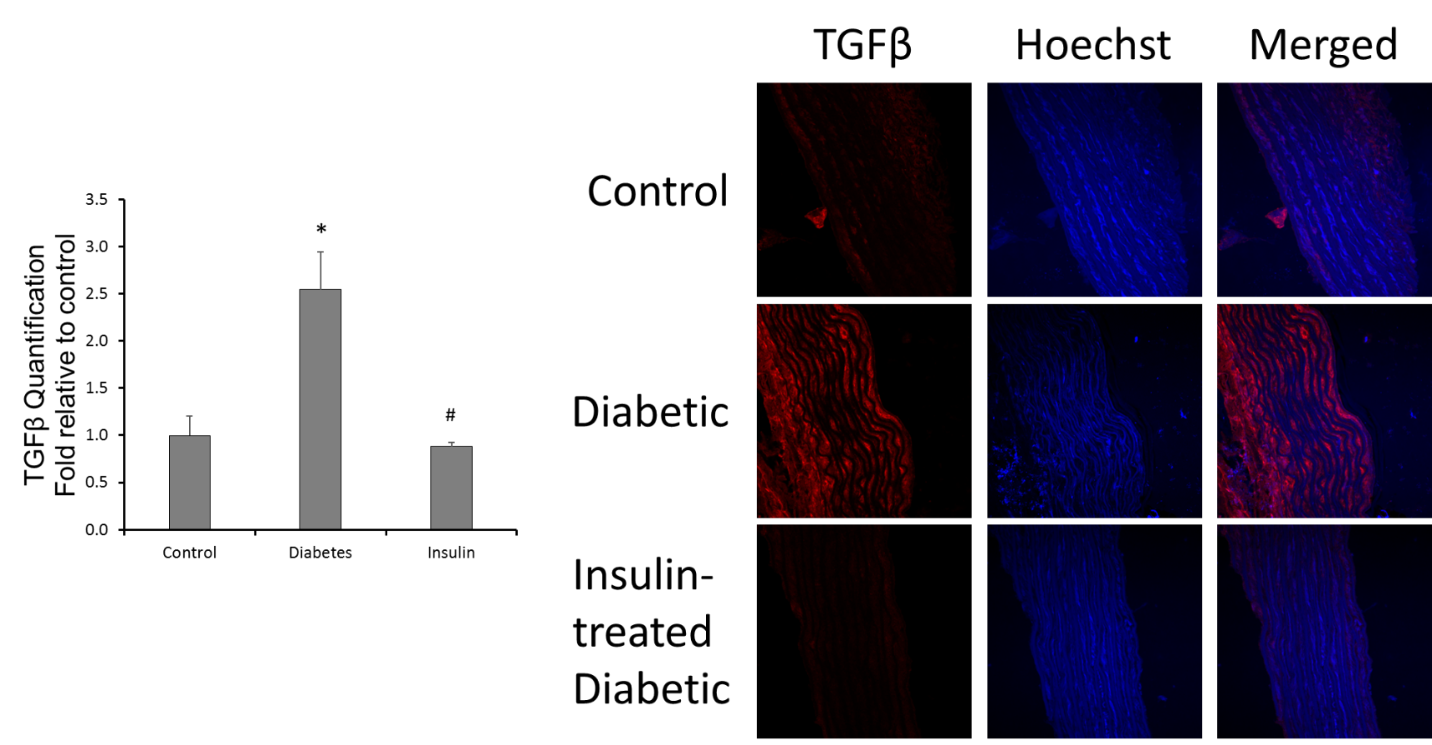


Figure B


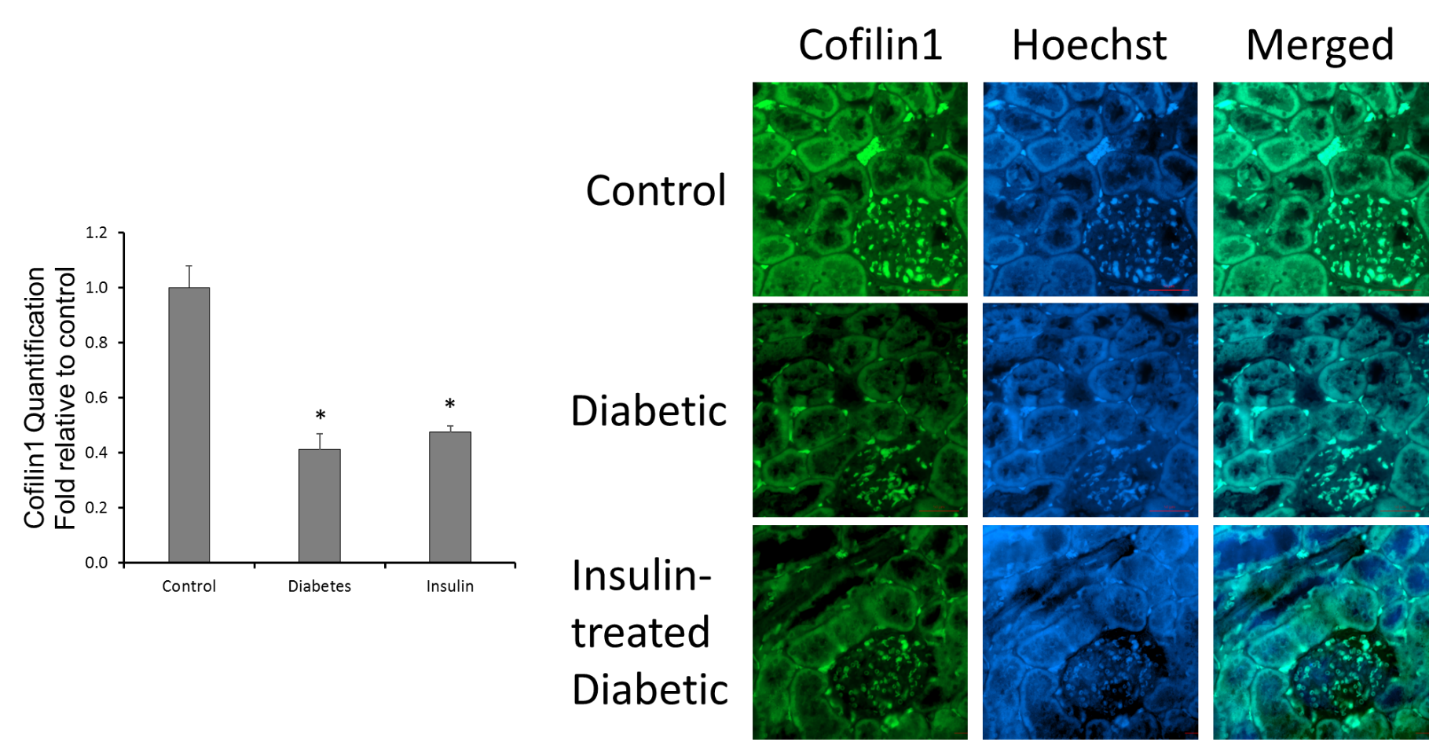


Figure C
